# Fenugreek Seed Powder Attenuates Lead-Induced Hepatic Injury and Renal Dysfunction in Male Mice Co-Exposed to Escalating Lead Doses

**DOI:** 10.3390/cimb48070650

**Published:** 2026-06-24

**Authors:** Muhammad Imran, Nosheen Mushtaq, Safdar Hussain

**Affiliations:** 1 Centre for Applied Molecular Biology (CAMB), University of the Punjab, Lahore, Punjab 53700, Pakistan; 2 Faculty of Allied Health Sciences, Lahore University of Biological and Applied Sciences, Lahore, Punjab 53500, Pakistan; nosheen.mushtaq@ubas.edu.pk

**Keywords:** lead toxicity, hepatorenal injury, fenugreek (*Trigonella foenum-graecum*), oxidative stress, inflammation, flavonoid C-glycosides, LC–DAD–ESI–MS/MS

## Abstract

Lead (Pb) induces oxidative stress, inflammation, and hepatorenal injury. We evaluated whether fenugreek (*Trigonella foenum-graecum*) seed powder (200 mg/kg) protects against subchronic Pb-acetate exposure in male albino mice. Sixty mice were randomized to six groups (n = 10): control (G1), fenugreek-only (G2), Pb 150 mg/kg (G3), and three co-exposure groups receiving fenugreek with Pb at 50, 100, and 150 mg/kg (G4–G6), gavaged daily for 8 weeks. LC–DAD–ESI–MS/MS of the seed batch tentatively identified 32 metabolites, dominated by flavonoid C-glycosides, luteolin dihydrogalloyl-glucosyl-pentosyl glucoside (15.90%), vicenin-3 (14.46%), vicenin-2 (9.66%), vicenin-1 (8.80%), kaempferol 7-O-rhamnosyl-glucoside (8.71%), with additional acylated phenolic conjugates. Pb exposure (G3) significantly reduced growth and intake, elevated serum ALT, AST, ALP, urea, and creatinine, raised blood Pb, and produced hepatic necrosis, vacuolation, and inflammation. Molecularly, Pb upregulated Nrf2, HO-1, SCD-1, TNF-α, and IL-6 and suppressed SOD-3. Fenugreek co-treatment attenuated all these changes across the three Pb doses, with greatest effect at the lowest Pb load (G4). Notably, fenugreek co-treatment reduced rather than further increased Nrf2 and HO-1 expression relative to Pb alone, a pattern most consistent with lowering the upstream oxidative stimulus rather than direct induction of these pathways. The seed’s polyphenolic profile—rich in vicenin-type C-glycosides and luteolin and kaempferol derivatives—offers a plausible chemical basis for the antioxidant, anti-inflammatory, and modest Pb-lowering effects observed; however, because whole seed powder was administered and metabolite identifications are tentative, these structure–activity relationships are presented as hypotheses for future bioactivity-guided fractionation rather than as demonstrated mechanisms. These preclinical findings support further investigation of fenugreek as a candidate dietary adjunct against environmental Pb exposure, contingent on protein-level validation, pharmacokinetic characterization, benchmarking against a standard chelator, and bioactivity-guided fractionation.

## 1. Introduction

Lead (Pb) is a non-essential heavy metal of major public health concern. Despite regulatory progress, human exposure continues through industrial activities, leaded gasoline residues, battery manufacture and recycling, ageing water-distribution systems, lead-based paints, contaminated soil, electronic waste, cosmetics, and traditional remedies, affecting populations in both high- and low-income countries [1].

Pb is toxic to multiple organs through several converging mechanisms: it induces oxidative stress, inhibits δ-aminolevulinic acid dehydratase (ALAD), disrupts calcium-dependent signaling, and competes with essential divalent cations, such as zinc and iron [2]. Children are particularly vulnerable; the World Health Organization has stated that no safe threshold for blood lead has been identified, and blood lead concentrations as low as 3.5 µg/dL have been linked to neurodevelopmental impairment in children. Chronic exposure leads to skeletal accumulation that can be remobilized during pregnancy, lactation, or osteoporosis [3], conferring increased risk of chronic kidney disease, hepatic dysfunction, cardiovascular events, and carcinogenesis. Despite progress in public health policy, Pb exposure continues to cause substantial morbidity and mortality worldwide, with primary toxicity driven by reactive oxygen species (ROS) generation, lipid peroxidation, inflammation, and disruption of energy metabolism [4]. In the liver specifically, Pb inhibits δ-aminolevulinic acid dehydratase (ALAD) and depletes thiol antioxidants, such as reduced glutathione, shifting the redox balance toward reactive oxygen species and driving lipid peroxidation of hepatocyte membranes; the consequent loss of membrane integrity underlies the leakage of cytosolic enzymes (ALT and AST) that signals hepatocellular injury. In the kidney, Pb preferentially accumulates in the proximal-tubule epithelium, where oxidative damage to tubular cells and mitochondria, together with Pb-induced glomerular and arteriolar changes, lowers the glomerular filtration rate and raises serum urea and creatinine. These convergent hepatic and renal mechanisms provide the rationale for the biochemical, histological, and molecular endpoints assessed in the present study.

Medicinal plants have emerged as candidate adjuncts against heavy-metal toxicity. Flavonoids, saponins, alkaloids, and phenolic acids can scavenge free radicals, chelate divalent metal ions, enhance endogenous antioxidant defenses, and modulate inflammatory signaling. Several preclinical studies have reported that fenugreek (*Trigonella foenum-graecum*) seed powder or extract attenuates Pb-induced hepatorenal toxicity in rodents [5,6], in part by restoring activity of endogenous antioxidant enzymes (SOD, CAT, and GPx). Beyond its redox effects, fenugreek may also influence Pb pharmacokinetics: recent reports describe reduced blood and tissue Pb concentrations in fenugreek-treated animals, consistent with intestinal absorption-limiting effects of the soluble galactomannan fiber fraction [6]. At the molecular level, fenugreek has been reported to lower pro-inflammatory cytokines (TNF-α and IL-6) and induce cytoprotective genes (Nrf2 and HO-1) [7], consistent with a multimodal protective profile.

Three specific gaps in the existing fenugreek-lead literature motivate the present study. First, most prior reports have evaluated fenugreek against a single Pb dose. Whether a fixed fenugreek dose remains protective across a range of Pb exposure intensities has not been systematically addressed. Second, the molecular signature of fenugreek’s protection has been examined for individual pathways but rarely integrated across redox-defense (Nrf2/HO-1/SOD-3), lipid-metabolic (SCD-1), and pro-inflammatory (TNF-α/IL-6) gene programs within the same animals. Third, whether the protective capacity of a fixed fenugreek dose is finite as Pb exposure intensity increases has received little explicit attention. Accordingly, we tested the hypothesis that a fixed dose of fenugreek seed powder (200 mg/kg) co-administered with lead acetate attenuates Pb-induced hepatic injury and renal functional impairment chiefly by lowering the upstream oxidative and inflammatory burden of Pb, and that the resulting protection is dose-limited, being greatest at the lowest Pb co-exposure and progressively overwhelmed as Pb dose increases, reflecting a finite protective capacity at a fixed phytochemical dose. We, therefore: (i) characterized subchronic Pb-acetate toxicity in male albino mice at 150 mg/kg over 8 weeks using a panel of physiological, biochemical, histological, and molecular endpoints; (ii) evaluated whether co-administration of fenugreek (200 mg/kg) attenuates this toxicity across three escalating Pb co-exposure doses (50, 100, and 150 mg/kg); and (iii) determined whether the magnitude of protection is dose-graded. In parallel, we characterized the polyphenolic composition of the fenugreek seed batch by LC–DAD–ESI–MS/MS to provide a defined phytochemical basis for the observed biological effects.

## 2. Materials and Methods

### 2.1. Experimental Animals

Male albino mice were obtained from the Institute of Pharmaceutical Sciences, University of Veterinary and Animal Sciences, Lahore, Pakistan. All experiments were conducted in strict accordance with global guidelines for the ethical use of animals in scientific research, including the Guide for the Care and Use of Laboratory Animals (National Research Council) and the ARRIVE (Animal Research: Reporting of in vivo Experiments) Guidelines. Ethical approval was obtained from the Institutional Review and Ethical Committee, University of the Punjab, Lahore, Pakistan (Approval No. 204/FIMS). Animals were housed under controlled environmental conditions (22 ± 2 °C, 55 ± 10% relative humidity, and 12 h light/dark cycle) with adequate ventilation and free access to feed and water. All efforts were made to minimize animal suffering, reduce the number of animals used, and ensure humane endpoints.

### 2.2. Experimental Design

**Figure 1:**
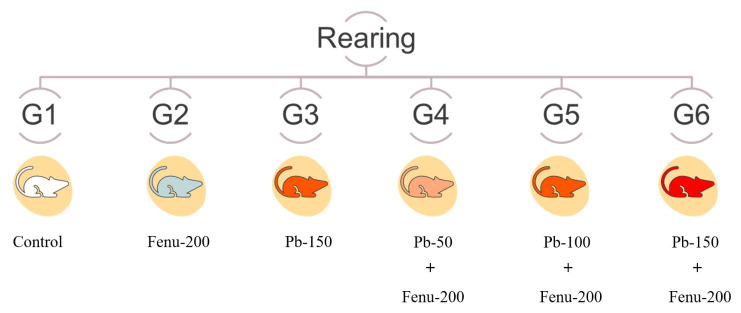
Mice are grouped by dose plan. All animals were randomly allocated to six groups (n = 10): G1, control (distilled water); G2, fenugreek-treated (200 mg/kg); G3, Pb-treated (150 mg/kg); G4, Pb (50 mg/kg) + fenugreek (200 mg/kg); G5, Pb (100 mg/kg) + fenugreek (200 mg/kg); and G6, Pb (150 mg/kg) + fenugreek (200 mg/kg).

Sixty healthy male albino mice (Age 6–8 weeks and weight 22–28 g) were randomly allocated to six groups (n = 10 per group): G1, the control receiving distilled water; G2, fenugreek 200 mg/kg only; G3, lead acetate 150 mg/kg only; G4, lead acetate 50 mg/kg + fenugreek 200 mg/kg; G5, lead acetate 100 mg/kg + fenugreek 200 mg/kg; and G6, lead acetate 150 mg/kg + fenugreek 200 mg/kg. All treatments were administered once daily by oral gavage for eight weeks to model subchronic environmental Pb exposure. The Pb-acetate doses (50, 100, 150 mg/kg) were chosen to span the range of subchronic exposure intensities established in published rodent toxicology to produce graded biochemical, histological, and molecular effects without acute lethality [2,3,4]. The 150 mg/kg dose corresponds to the high-dose Pb-only arm reported by Andjelkovic et al. [3] and adjacent comparative studies. The eight-week treatment duration was selected to capture cumulative subchronic effects on hepatic and renal endpoints, consistent with prior fenugreek heavy-metal protection studies in rodents [5,6,8]. The animals were housed in a routine lab environment (22 ± 2 °C, 55 ± 10% humidity, and 12 h light/dark cycle) with free access to food and water. Prior to the start of dosing, all animals were acclimatized to laboratory conditions for 7 days with free access to standard rodent chow and water. Body weight was recorded once every two weeks (W0, W2, W4, W6, and W8), and food and water consumption were recorded daily per cage and expressed per mouse per day throughout the eight-week period. All mice were observed daily for standardized clinical and humane-endpoint indicators, including body condition, posture, coat condition, activity, signs of pain or distress, and any morbidity or mortality; predefined humane endpoints (e.g., ≥20% body-weight loss, moribund condition, or inability to access food or water) prompted euthanasia (Figure 1).

### 2.3. Diet Preparation

Lead acetate trihydrate (analytical grade) was freshly dissolved in distilled water to provide Pb-acetate dosing solutions delivering 50, 100, or 150 mg lead per kg body weight. Fenugreek (*Trigonella foenum-graecum* L.) seeds were obtained from a local market in Kasur, Pakistan, and authenticated by a qualified botanist at the Department of Botany, University of the Punjab. The seeds were cleaned of foreign matter, shade-dried at room temperature for two days, and ground to a fine powder using a mechanical grinder. The powder was passed through a 40-mesh sieve, stored in airtight containers at 4 °C until use, and freshly suspended in distilled water immediately before each gavage to give a dose of 200 mg/kg body weight. The 200 mg/kg fenugreek dose was selected on the basis of prior rodent studies demonstrating efficacy of similar fenugreek seed powder doses against heavy-metal and chemical toxicity without observable adverse effects [3,5,8].

### 2.4. Sample Collection for HPLC

Phytochemical characterization of the fenugreek seed batch was performed by LC–DAD–ESI–MS/MS in negative-ion mode. Seeds were extracted using a Dionex ASE 200 system (methanol/water 50:50 *v*/*v*, 80 °C, 1500 psi, and 3 cycles). Chromatographic separation used a Waters Nova-Pak C18 column (4.9 × 250 mm, 4 µm) with a gradient of water/acetic acid (solvent A) to acetonitrile/acetic acid (solvent B) at 1 mL/min. UV detection was performed at 280, 310, 330, and 360 nm. ESI-MS was scanned at *m*/*z* 100–3000 in negative-ion mode (capillary voltage 4000 V; N_2_ drying gas 10 L/min, 350 °C; and nebulizer pressure 55 psi). Thirty-two metabolites were tentatively identified on the basis of retention time, UV λmax, [M − H]^−^ ion, and MS/MS fragmentation, supported by published reference data [9,10,11]. Relative abundances were determined from integrated peak areas at 280 nm.

### 2.5. Blood Sample Collection for Biochemical, Histological, and Molecular Analyses

At the end of the eight-week treatment period, mice were fasted overnight and then euthanized by intraperitoneal injection of sodium pentobarbital (40–60 mg/kg), in accordance with American Veterinary Medical Association (AVMA) guidelines. Blood was collected by cardiac puncture into plain serum tubes; please note that hemolyzed samples were excluded. Samples were allowed to clot at room temperature, centrifuged at 3500× *g* for 10 min, and the resulting serum stored at −80 °C until biochemical analysis. The liver and kidneys were excised, rinsed in ice-cold phosphate-buffered saline (PBS), and processed for histology and molecular analysis as described below.

### 2.6. Biochemical Assays

Liver and kidney function indicators were measured using Sigma-Aldrich (Burlington, MA, USA) test kits (ALT Kit, MAK052; AST Kit, MAK055; ALP Kit, MAK089; Urea Kit, UR2210; and Creatinine Kit, CR510).

### 2.7. Lead Concentration AAS

Whole-blood samples were digested in a nitric acid:perchloric acid (6:1, *v*/*v*) mixture and analyzed for total lead using a Varian Spectra A250 flame atomic absorption spectrophotometer at λ = 217.0 nm, following the method of Trzcinka-Ochocka et al. [11].

### 2.8. Histopathology

Liver tissue was rapidly excised after euthanasia, fixed in 10% neutral-buffered formalin for 24–48 h, dehydrated through graded ethanol, cleared in xylene, and embedded in paraffin. Sections of 5 µm thickness were cut on a rotary microtome, mounted on glass slides, and stained with hematoxylin and eosin (H&E). The slides were examined by light microscopy by two independent observers blinded to treatment group, who scored each section for hepatocellular degeneration, necrosis, inflammatory-cell infiltration, and cytoplasmic vacuolation on a 0–4 semi-quantitative scale (0 = absent, 1 = minimal, 2 = mild, 3 = moderate, and 4 = marked). Scores were averaged across three fields per animal and across all animals per group, with discrepancies resolved by consensus. Inter-rater reliability was assessed using Cohen’s κ statistic. Agreement between the two observers was substantial for the unweighted scores (Cohen’s κ = 0.69; 95% CI 0.38–0.92) and near-perfect once the ordinal nature of the 0–4 scale was taken into account, as all disagreements were by a single grade (quadratic-weighted κ = 0.91; 95% CI 0.73–0.98), and 95% confidence intervals were obtained by bootstrap resampling (10,000 iterations).

### 2.9. Gene Expression Analysis

Hepatic mRNA expression of redox-related (Nrf2, SOD-3, HO-1), metabolic (SCD-1), and pro-inflammatory (TNF-α, IL-6) genes was quantified by quantitative real-time PCR (qPCR). These six genes were selected to capture three mechanistic axes of Pb-induced toxicity: (i) Nrf2 and its downstream target HO-1 represent the master cytoprotective antioxidant response activated under Pb-induced ROS stress [7,9]; (ii) SOD-3 (extracellular superoxide dismutase) is a key enzymatic scavenger, whose downregulation indicates antioxidant-defense failure [11]; (iii) SCD-1 (stearoyl-CoA desaturase 1) reports Pb-induced lipid metabolic dysregulation [11]; and (iv) TNF-α and IL-6 are the principal NF-κB-driven pro-inflammatory cytokines associated with Pb toxicity (Table 1) [10,12,13]. Total RNA was extracted from liver tissue using TRIzol reagent (Invitrogen, Waltham, MA, USA), following the manufacturer’s protocol. RNA integrity and concentration were verified spectrophotometrically. First-strand cDNA was synthesized from 1–2 μg total RNA in a 20-μL reaction using a Revert Aid First Strand cDNA Synthesis Kit (K1622; Thermo Fisher Scientific, Waltham, MA, USA), with cycling of 25 °C/5 min, 42 °C/60 min, and 85 °C/5 min. qPCR was performed on an Applied Biosystems QuantStudio 3 system using PowerUp SYBR Green Master Mix (A25742; Thermo Fisher Scientific) in 20-μL reactions containing 1 μL cDNA template and 250–500 nM of each primer. Cycling conditions were 95 °C for 10 min, followed by 40 cycles of 95 °C for 15 s, and 60 °C for 1 min, with melt-curve analysis from 60 °C to 95 °C in 0.3 °C increments. Primers (Table 2) were designed in NCBI Primer-BLAST (https://www.ncbi.nlm.nih.gov/tools/primer-blast/ (accessed on 20 November 2023)) and checked for self-dimer formation using OligoCalc (http://biotools.nubic.northwestern.edu/OligoCalc.html (accessed on 20 November 2023)). Amplicon size was confirmed by 2% agarose gel electrophoresis and primer specificity by melt-curve analysis. The primers were designed against the mouse reference genome assembly GRCm39 (RefSeq assembly GCF_000001635.27); the corresponding genomic RefSeq accession numbers and melting temperatures are listed in Table 2. β-actin served as the endogenous reference. Each sample was assayed in technical triplicate. Relative mRNA expression was calculated by the 2^−ΔΔCt^ method, using the control group (G1) as the calibrator.

**Table 1:** Summary of potential genes associated with oxidative stress and inflammation.

Sr.	Gene	Reported Association	Reference
1.	Nrf2	Nrf2 gene is involved in regulating the antioxidant response, and has been found to play a role in protecting against lead-induced oxidative stress.	[14]
2.	TNF-α	A significant association between indicator of inflammation (higher serum TNF-α) in male subjects with blood lead ≥2.51 μg/dL.	[15]
3.	IL-6	IL-6 is a proinflammatory cytokine that influences Pb cytokine metabolism.	[16]
4.	SOD-3	Encodes an antioxidant enzyme essential in oxidative stress and inflammation.	[17]
5.	SCD1	Most significantly associated with oxidative stress, as reported in a study.	[17]
6.	HO-1	HO-1 is an antioxidant enzyme that plays a critical role in protecting cells against oxidative stress.	[18]

**Table 2:** Primer sequences used for each gene in qPCR.

Sr.	Gene	Amplicon Size	Sequence (5′→3′) Forward (Tm, °C; NCBI RefSeq Accession)	Sequence (5′→3′) Reverse (Tm, °C)
1	Nrf2	99	TCTGTTTTTCCAGCTCATAGTCCT (Tm 59.7 °C; RefSeq NC_000068.8)	TGATTGACATCCTTTGGAGGC (Tm 60.0 °C)
2	TNF-α	134	TGGGTGAGGAGCACGTAGT (Tm 58.0 °C; RefSeq NC_000083.7)	GCCAACGCCCTCCTGG (Tm 57.1 °C)
3	IL-6	112	TTGTGCAATGGCAATTCTGAT (Tm 57.1 °C; RefSeq NC_000071.7)	GATTATATCCAGTTTGGTAGCATCC (Tm 59.1 °C)
4	SOD-3	190	CTGTGGCTCTGTCACCATGT (Tm 58.0 °C; RefSeq NC_000071.7)	GATTGCATGCATCTCGGCAG (Tm 60.1 °C)
5	SCD1	226	CACCCAGGGAAACCAGGATA (Tm 58.7 °C; RefSeq NC_000085.7)	TACTACAAGCCCGGCCTCC (Tm 61.1 °C)
6	HO-1	264	GTGATGGCTTCCTTGTACCAT (Tm 58.0 °C; RefSeq NC_000074.7)	GTAGCGGGTATATGCGTGGG (Tm 60.4 °C)
7	β-actin	171	CCAGGTCCAGACGCAGGAT (Tm 61.4 °C; RefSeq NC_000071.7)	TTGAGACCTTCAACACCCCA (Tm 58.8 °C)

### 2.10. Statistical Analysis

All data were analyzed using GraphPad Prism (version 10; GraphPad Software, San Diego, CA, USA) and are presented as mean ± SEM (n = 10 per group) unless otherwise stated. Normality was assessed by the Shapiro–Wilk test and homogeneity of variance by the Brown–Forsythe and Bartlett’s tests. Each endpoint was first evaluated with a single omnibus test of the overall treatment effect: one-way ANOVA when normality and variance homogeneity held; Welch’s ANOVA when variances were heterogeneous (Brown–Forsythe or Bartlett’s, *p* < 0.05 for AST, ALP, creatinine, blood lead, Nrf2, HO-1, and SCD-1); and the Kruskal–Wallis test for non-normal data (histopathology scores). The specific omnibus test and post hoc procedure actually applied to each endpoint, determined by these assumption checks on the underlying data, are reported in the corresponding figure legend and in Supplementary Table S1. To keep the analysis aligned with the study aims and to avoid the inflated multiplicity and poor readability of reporting all fifteen possible pairwise contrasts, post hoc testing was restricted to a pre-specified set of purpose-driven comparisons, each mapped to a specific aim: (i) lead toxicity—Pb-only (G3) versus the control (G1); (ii) intrinsic safety of fenugreek—fenugreek-only (G2) versus the control (G1); (iii) protective effect of fenugreek—each co-exposure group versus the Pb-only reference (G4, G5, and G6 versus G3), with G6 versus G3 (both 150 mg/kg Pb) constituting the matched-dose comparison; and (iv) residual injury after protection—each co-exposure group versus the control (G4, G5, and G6 versus G1). Comparisons against the common control (G1) were made with Dunnett’s test, and the co-exposure versus G3 contrasts with Šídák’s correction. Where variances were heterogeneous, the corresponding Games–Howell comparisons were limited to the same pre-specified set. Contrasts not relevant to these aims (in particular, comparisons among the co-treatment groups G4–G6) were regarded as exploratory and are not emphasized. A two-tailed adjusted *p* < 0.05 was considered significant, and significance is denoted uniformly throughout the figures and text as * *p* < 0.05, ** *p* < 0.01, *** *p* < 0.001, and **** *p* < 0.0001 (ns, not significant). Effect sizes (η^2^ or R^2^) and 95% confidence intervals are reported where appropriate.

## 3. Results

Throughout this section, statistical significance refers to the pre-specified, aim-relevant comparisons defined in Section 2.10: lead toxicity (G3 vs. G1), the intrinsic safety of fenugreek (G2 vs. G1), the protective effect of fenugreek (G4, G5, and G6 vs. the Pb-only group G3, including the matched-dose comparison G6 vs. G3), and residual injury after protection (G4, G5, and G6 vs. G1). Comparisons among the co-treatment groups (G4–G6) are exploratory and are noted only where directly relevant.

### 3.1. HPLC Component Analysis

**Figure 2:**
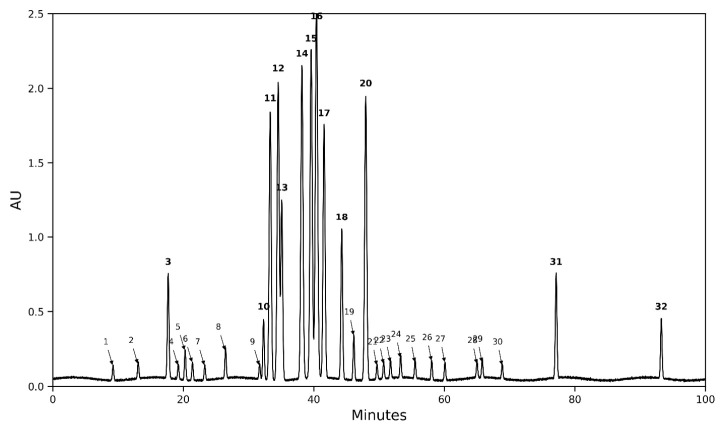
An HPLC chromatogram of the studied fenugreek crude seeds recorded at 280 nm.

The fenugreek seed extract was analyzed by LC–DAD–ESI–MS/MS in negative-ion mode. The UV chromatogram at 280 nm (Figure 2) showed 32 chromatographic peaks corresponding to phytochemical constituents identified on the basis of retention time, UV λmax, [M − H]^−^ ion, MS/MS fragmentation, and comparison with the published literature [19].

The HPLC separation yielded 32 well-resolved peaks. Compound identifications were based on retention time, online UV–Vis spectra, ESI mass spectra, and published reference data [19].

The online UV–Vis spectra of the identified phenolics were consistent with flavone and flavonol glycosides and phenolic acids. Most flavonoids displayed characteristic UV absorption maxima at 232–270 and 316–346 nm [20]. Several peaks also showed UV absorption characteristic of hydroxycinnamic acid derivatives.

Molecular ions and characteristic fragmentation patterns enabled tentative identification of the resolved compounds. Higher ESI cone voltages enhanced fragmentation and revealed the presence of acyl substituents. Only peaks producing observable [M − H]^−^ ions are reported in Table 3; several minor peaks were below the threshold for reliable structural assignment.

Several flavonoids carried C- or O-hexoside, pentoside, or acyl substituents. The fragmentation patterns observed are consistent with prior reports indicating that flavones and flavonols are typically glycosylated at the 7-hydroxyl position, and that C-glycosides are attached at C-6 or C-8 [21,22]. The type of O- or C-glycosidic linkage and the positional isomerism of flavone and flavonol glycosides were determined from the relative intensities of product ions [21,23]. Many flavonoid glycosides exhibited 1→2 and 1→6 interglycosidic linkages in their sugar moieties [24,25].

LC–ESI–MS/MS analysis tentatively identified 32 phytochemical constituents in the extract, comprising flavone C-glycosides, phenolic acid derivatives, and highly acylated flavonoid conjugates. These classes are well-known to possess antioxidant, anti-inflammatory, hepatoprotective, and chemopreventive activities [26].

Early-eluting peaks (1–8; Rt 9.25–26.48 min) were predominantly phenolic acid conjugates and hydroxycinnamic acid derivatives. Peak 1 was tentatively identified as tricaffeoyl-glucosyl-glucoside; Peak 2 as a tricaffeoyl-hydroxyferulic acid derivative; Peak 3 as a dihydrogallic acid derivative; Peak 4 as a disynapoyl-hydroferuloyl-feruloyl-hydrocaffeic acid; Peak 5 as a galloyl-coumaric acid pentoside; Peak 6 as caffeoyl-coumaroyl-quinic acid; and Peak 7 as dicaffeoyl-protocatechuic acid diglucoside. These compounds typically exhibit antioxidant activity through their conjugated aromatic systems and multiple hydroxyl substituents [27].

Between Rt 31.70 and 68.86 min, flavone C-glycosides dominated the chromatogram. Peaks 9, 11, and 13 corresponded to vicenin-2 (apigenin 6,8-di-C-hexoside) and its positional isomers, all sharing the molecular ion at *m*/*z* 593 and the diagnostic fragments at *m*/*z* 473, 383, and 353. These cross-ring cleavages, characteristic of C-glycosyl flavones rather than glycosidic bond dissociation, are typical of vicenin-type LC–MS/MS spectra [28].

Peaks 14 and 17 ([M − H]^−^ at *m*/*z* 563) were tentatively identified as vicenin-3 and vicenin-1, respectively. These structural isomers differ in the position of pentosyl and glucosyl substitution on the apigenin skeleton. The co-occurrence of multiple vicenin isomers reflects the diversity of flavone-biosynthetic intermediates in the seed. Vicenin derivatives are reported to possess anti-inflammatory and antioxidant activities in numerous medicinal plants [28].

Several heavily substituted apigenin derivatives were also detected. Peak 10 corresponded to apigenin 6-C-glucosyl 8-C-(2″-O-dihydroferuloyl)-glucoside, and Peak 12 to a related vicenin derivative. Peaks 18, 20, 21, 22, 23, 26, and 27 corresponded to galloylated, hydroxyferuloylated, methoxygalloylated, and rhamnosylated apigenin glycosides. Galloyl and feruloyl acyl substituents introduce additional hydroxyl groups that stabilize radicals and enhance antioxidant potential [28].

Luteolin derivatives were prominent in the extract. Peaks 15 and 16 showed the highest UV absorbance (AU 2.2 and 2.5, respectively) and were tentatively identified as luteolin 7-O-[6″-dihydrogalloyl]-glucosyl-8-C-pentosyl-glucoside derivatives. The UV absorption maxima at 346–348 nm were consistent with luteolin-based flavones. Luteolin and its glycosides possess an ortho-dihydroxyl B-ring that supports both metal chelation and radical scavenging, and recent reviews have highlighted their anti-inflammatory, anticancer, antimicrobial, and hepatoprotective activities [26].

Peaks 25, 28, and 30 were tentatively identified as luteolin 7-O-(2″-galloyl)-glucosyl 6-C-(2‴-pentosyl)-rhamnoside derivatives.

Owing to greater hydrophobicity, kaempferol derivatives eluted later. Kaempferol 7-O-(6″-galloyl)-glucosyl-6-C-(2‴-pentosyl)-rhamnoside and kaempferol 7-O-rhamnosyl-(1→2)-glucoside were tentatively assigned to Peaks 24 and 31, respectively. Peak 32 (Rt 93.23 min; [M − H]^−^ 1133) was tentatively identified as the structurally complex kaempferol 7-O-(2‴,6‴,2″-malonyl)-rhamnosyl-diglucosyl-3-O-(6″″′-rhamnosyl) derivative. These glycosides have reported anti-inflammatory, antioxidant, and cardioprotective activities [27].

In summary, LC–ESI–MS/MS analysis revealed that the fenugreek seed extract contained predominantly flavone C-glycosides and acylated flavonoid derivatives, with apigenin, luteolin, and kaempferol glycosides and hydroxycinnamic acid conjugates as the principal chemical classes. The diversity of glycosylation and acylation patterns supports a broad antioxidant and pharmacological potential for this extract [27].

A quantitative LC–MS study of fenugreek seed extract showed a wide range of phytochemical abundances, from 0.04% to 15.90% of the integrated peak area (Table 4).

Luteolin 7-O-[6″-dihydrogalloyl]-glucosyl-8-C-pentosyl-(1→2)-glucoside was the most abundant compound in the extract (Peak 16; 15.90%), followed by apigenin 8-C-xyloside-6-C-glucoside (vicenin-3; Peak 14; 14.46%) and an apigenin 7-O-(2″-dihydrogalloyl)-rhamnosyl-6-C-(2‴-pentosyl)-glucoside derivative (Peak 20; 12.10%).

Other major constituents included apigenin 6,8-di-C-glucoside (vicenin-2; Peak 11; 9.66%), apigenin 6-C-xyloside-8-C-glucoside (vicenin-1; Peak 17; 8.80%), kaempferol 7-O-rhamnosyl-(1→2)-glucoside (Peak 31; 8.71%), apigenin 7-O-(2″-dihydrogalloyl)-rhamnosyl derivative (Peak 18; 6.56%), and apigenin 6,8-di-C-hexoside (vicenin-2 isomer; Peak 13; 5.67%).

Together, these constituents represent the dominant flavonoid fraction of the extract and are composed mainly of apigenin-, luteolin-, and kaempferol-based glycosides. Compounds present at moderate abundance included luteolin 7-O-[6″-dihydrogalloyl]-glucosyl-8-C-pentosyl-(1→6)-glucoside (Peak 15; 3.78%), a dihydrogallic acid derivative (Peak 3; 3.14%), a kaempferol 7-O-(2‴,6‴,2″-malonyl)-rhamnosyl-diglucosyl derivative (Peak 32; 2.50%), and apigenin 8-C-rhamnoside-6-C-glucoside (Peak 19; 1.16%).

These compounds contribute to the secondary phytochemical pool of the extract and represent structurally diverse flavonoid and phenolic-acid derivatives.

A further 17 constituents were detected at low abundance (<1%) but contribute to the overall chemical diversity of the extract. These include tricaffeoyl-glucosyl-glucoside (Peak 1; 0.18%), tricaffeoyl-hydroxyferulic acid (Peak 2; 0.19%), disynapoyl-hydroferuloyl-feruloyl-hydrocaffeic acid (Peak 4; 0.47%), galloyl-coumaric acid pentoside (Peak 5; 0.44%), caffeoyl-coumaroyl-quinic acid (Peak 6; 0.24%), dicaffeoyl-protocatechuic acid diglucoside (Peak 7; 0.36%), and a range of acylated apigenin, luteolin, and kaempferol glycosides (Peaks 9, 10, 21–30; see Table 3 for full details). The full quantitative composition is reported in Table 4.

The extract was dominated by flavonoid C-glycosides—principally apigenin- and luteolin-derived compounds—which together accounted for the largest fraction of the total integrated peak area. Phenolic-acid conjugates and highly acylated flavonoids were present at lower individual abundance but contributed substantially to the structural diversity of the metabolite pool.

A limited number of structurally stable flavonoid glycosides—particularly vicenin-type C-glycosides and luteolin derivatives—dominate the phytochemical composition, while a larger number of minor phenolics contribute to overall chemical complexity and to potential synergistic biological activity.

### 3.2. Response of Treatment on Body Weight and Food and Water Intake

**Figure 3:**
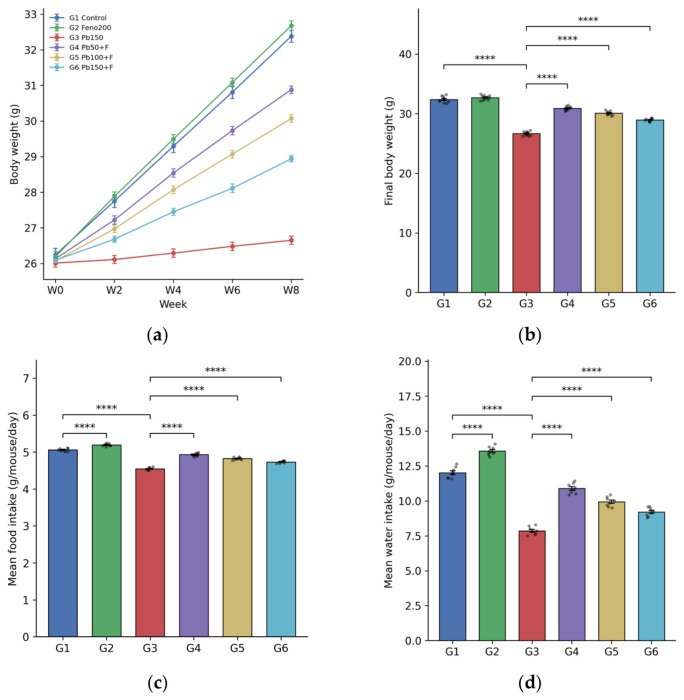
Weight, diet, and water. (**a**) Average 8-week mouse weight. At weeks 0, 2, 4, 6, and 8, 60 mice were assessed for their mean body weight (±SD). Each point indicates the mean body weight at the stated time, with the error bars showing the standard deviation. A modest weight gain was noticed during the trial. (**b**) Final body weight (g) per group at Week 8. All groups, except G3 (Pb 150 mg/kg), gained weight relative to the baseline; fenugreek co-treatment (G4–G6) partially restored body-weight gain compared with Pb-only exposure (G3). (**c**) Pb treatment (G3) significantly reduced food intake compared with the control (G1, ns) and fenugreek (G2, **** *p* < 0.0001). Fenugreek (G2) alone increased food intake compared with the control and Pb (*** *p* < 0.001). Among the co-treatment groups, food intake in G4 was significantly higher than in Pb-only (G3; * *p* = 0.0201), whereas G5 and G6 did not differ significantly from G3 (ns). (**d**) Pb treatment (G3) decreased water intake compared with the control (G1, ns), but fenugreek alone (G2) increased water intake compared with both the control (**** *p* < 0.0001) and Pb groups (**** *p* < 0.0001). The co-treatment groups (G4–G6) showed modest recovery, with G4 significantly recovering (*p* < 0.05–0.01 vs. G2 and G3), whereas G5 and G6 did not statistically differ (ns). The data are presented as the mean ± SEM (n = 10). The tests were applied per endpoint, as defined in Section 2.10: body weight and water intake by one-way ANOVA with the pre-specified Dunnett’s/Šídák comparisons, and food intake by the Kruskal–Wallis test with Dunn’s comparisons; only the pre-specified, aim-relevant comparisons are shown. Significance: * *p* < 0.05, ** *p* < 0.01, *** *p* < 0.001, **** *p* < 0.0001. Ns = non-significant. The omnibus test and post hoc procedure for each panel are reported in Supplementary Table S1.

Starting the study (Week 0), the mice in all groups had a mean weight of 26.12 ± 0.39 g (n = 60). During the 8-week study, the animal cohort gained weight gradually, reaching 30.27 ± 2.11 g by Week 8 (Table 5 & Figure 3a).

Repeated-measures ANOVA over the 8-week treatment period revealed significant within-subject consistency across time points (*p* < 0.0001). Table 6 and Figure 3b show the final body-weight descriptive statistics. The control and fenugreek-only groups had significantly higher mean final body weights (32.38 ± 0.52 g and 32.68 ± 0.41 g, respectively; *p* < 0.0001) compared with Pb-only (G3).

The experimental treatments significantly impacted the mice’s daily food intake (*p* < 0.0001). Post hoc analysis under the pre-specified comparisons (Section 2.10) revealed group differences (Figure 3c).

Final body weight and daily food and water intake were each strongly affected by treatment (full statistics in Supplementary Table S1). Pb-only depressed all three relative to the control, and fenugreek co-treatment significantly restored them across Pb doses (body weight: n = 10 per group; and food and water intake: n = 8 weekly cage-mean measurements per group). Final body weight: one-way ANOVA F(5,54) = 352.9, *p* < 0.0001, η^2^ = 0.97; Pb-only vs. control (G3 vs. G1) **** (*p* < 0.0001); fenugreek-only vs. control (G2 vs. G1) ns (*p* = 0.2813); co-treatment vs. Pb-only (Dunnett’s (vs. G1) + Šídák (vs. G3)) G4 **** (*p* < 0.0001), G5 **** (*p* < 0.0001), G6 **** (*p* < 0.0001). Food intake: one-way ANOVA F(5,42) = 328.6, *p* < 0.0001, η^2^ = 0.98; Pb-only vs. control (G3 vs. G1) **** (*p* < 0.0001); fenugreek-only vs. control (G2 vs. G1) **** (*p* < 0.0001); co-treatment vs. Pb-only (Dunnett’s (vs. G1) + Šídák (vs. G3)) G4 **** (*p* < 0.0001), G5 **** (*p* < 0.0001), G6 **** (*p* < 0.0001). Water intake: one-way ANOVA F(5,42) = 291.4, *p* < 0.0001, η^2^ = 0.97; Pb-only vs. control (G3 vs. G1) **** (*p* < 0.0001); fenugreek-only vs. control (G2 vs. G1) **** (*p* < 0.0001); co-treatment vs. Pb-only (Dunnett’s (vs. G1) + Šídák (vs. G3)) G4 **** (*p* < 0.0001), G5 **** (*p* < 0.0001), G6 **** (*p* < 0.0001).

The Pb-only group (G3, Pb 150 mg/kg) had substantially lower food intake (4.548 ± 0.036 g/mouse/day) compared with the control group (G1, 5.059 ± 0.039 g/mouse/day), consistent with Pb-induced appetite suppression. Co-administration of fenugreek partially attenuated this anorexic effect. Food intake in G4 was higher than in Pb-150 (G3, *p* = 0.0201), but not statistically different from the control (G1, *p* > 0.9999). The Pb100–fenugreek and Pb150–fenugreek groups had higher food intake (G5: 4.821 ± 0.035 g/mouse/day; G6: 4.731 ± 0.029 g/mouse/day) than the Pb150-only group (G3, *p* = 0.4876 and *p* > 0.9999, respectively) (Table 6 and Figure 3c).

The treatments significantly altered daily water consumption among the groups (*p* < 0.0001), and group-specific differences were identified by post hoc analysis (Table 5 and Figure 3d).

Lead exposure markedly reduced water consumption. The Pb150 group (G3) had the lowest mean daily water intake (7.858 ± 0.291 mL/mouse/day), significantly lower than all of the other groups: the control (G1: 12.02 ± 0.395 mL/mouse/day; *p* < 0.0001), fenugreek-200 (G2: 13.57 ± 0.313 mL/mouse/day; *p* < 0.0001), Pb50–fenugreek (G4: 10.89 ± 0.369 mL/mouse/day; *p* = 0.0013), Pb100–fenugreek (G5: 9.938 ± 0.337 mL/mouse/day; *p* = 0.0325), and Pb150–fenugreek (G6: 9.210 ± 0.313 mL/mouse/day; *p* = 0.2850 [ns]).

Co-administration of fenugreek seed powder partially restored water consumption in Pb-exposed mice. Pb50–fenugreek (G4) consumed more water than Pb150-only (G3) and, on the weekly intake data, remained significantly below the control (G1, *p* < 0.0001). Pb100–fenugreek (G5) and Pb150–fenugreek (G6) had larger intakes than Pb150 (G3) but remained significantly below the control (G1; *p* < 0.0001 for both) and fenugreek-alone (G2).

### 3.3. Biochemical Findings

**Figure 4:**
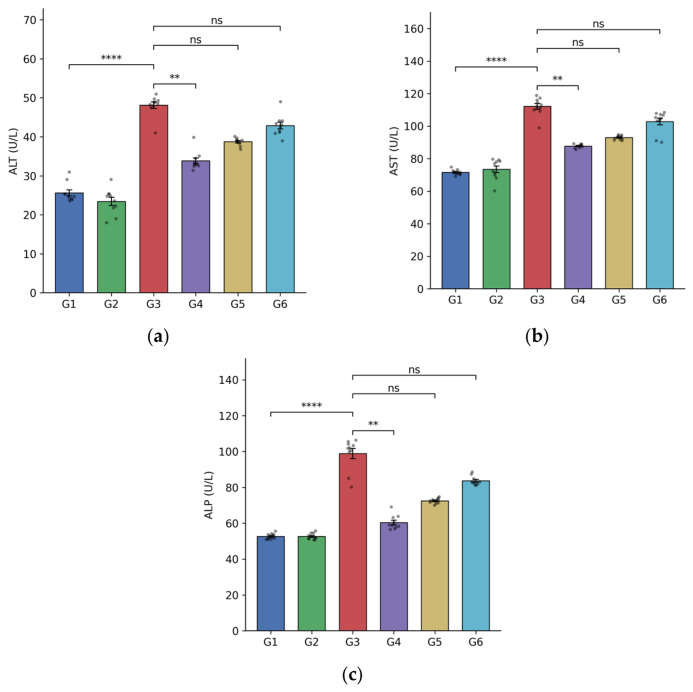
Mice liver analysis. (**a**) Six groups had ALT (U/L) tests. G1 and G2 had equal ALT activity (ns), demonstrating liver health. Pb-150 significantly increased ALT (**** *p* < 0.0001, **** *p* < 0.0001 vs. G1 and G2), indicating hepatocellular injury. Under the pre-specified focused comparisons, fenugreek significantly lowered ALT relative to Pb-150 only at the lowest Pb co-exposure (G4 vs. G3, ** *p* = 0.002); G5 and G6 did not differ significantly from G3 (ns). All co-treatment groups remained above G1. (**b**) Serum aspartate aminotransferase (AST) levels were significantly altered among experimental groups. G3 had the most AST activity, followed by G6, G5, and G4, whereas G1 and G2 had equal baseline levels. The data are shown as the mean ± SD (n = 10 per group). The omnibus test and post hoc procedure for AST follow the data-driven scheme in Section 2.10 (Kruskal–Wallis with Dunn’s test) and are reported in Supplementary Table S1. Under the focused comparisons, AST was significantly lowered relative to Pb-only at the lowest Pb co-exposure (G4 vs. G3, ** *p* = 0.001); G5 and G6 did not differ significantly from Pb-only (ns). (**c**) Serum alkaline phosphatase (ALP) levels showed significant variation among all of the experimental groups. Group 3 exhibited the highest ALP activity, followed by Group 6, Group 5, and Group 4, whereas Groups 1 and 2 showed the lowest and comparable baseline levels. The data are presented as the mean ± SD (n = 10 per group). Statistical analysis followed the data-driven scheme in Section 2.10 (Kruskal–Wallis with Dunn’s test; see Supplementary Table S1). Under the focused comparisons, ALP was significantly lowered relative to Pb-only at the lowest Pb co-exposure (G4 vs. G3, ** *p* = 0.001); G5 and G6 did not differ significantly from Pb-only (ns). Statistical significance is indicated uniformly as * *p* < 0.05, ** *p* < 0.01, *** *p* < 0.001, and **** *p* < 0.0001 (ns, not significant), as defined in Section 2.10. The omnibus test and post hoc procedure for each panel are reported in Supplementary Table S1.

Serum ALT levels varied considerably among the six experimental groups (Figure 4a), as detailed in the focused analysis below and in Supplementary Table S1.

Each liver-enzyme endpoint showed a strong overall treatment effect (full statistics in Supplementary Table S1), with Pb-only markedly elevated versus the control, and fenugreek co-treatment lowering enzyme levels the most at the lowest Pb co-exposure. ALT: Kruskal–Wallis H = 54.2, *p* < 0.0001, ε^2^ = 0.91; Pb-only vs. control (G3 vs. G1) **** (*p* < 0.0001); fenugreek-only vs. control (G2 vs. G1) ns (*p* = 0.6726); and co-treatment vs. Pb-only (Dunn (Holm)) in G4 ** (*p* = 0.0025), G5 ns (*p* = 0.0578), and G6 ns (*p* = 0.6482). AST: Kruskal–Wallis H = 54.2, *p* < 0.0001, ε^2^ = 0.91; Pb-only vs. control (G3 vs. G1) **** (*p* < 0.0001); fenugreek-only vs. control (G2 vs. G1) ns (*p* = 0.7392); and co-treatment vs. Pb-only (Dunn (Holm)) in G4 ** (*p* = 0.0011), G5 ns (*p* = 0.1088), and G6 ns (*p* = 0.3702). ALP: Kruskal–Wallis H = 55.1, *p* < 0.0001, ε^2^ = 0.93; Pb-only vs. control (G3 vs. G1) **** (*p* < 0.0001); fenugreek-only vs. control (G2 vs. G1) ns (*p* = 1); and co-treatment vs. Pb-only (Dunn (Holm)) in G4 ** (*p* = 0.0014), G5 ns (*p* = 0.0643), and G6 ns (*p* = 0.6609).

ALT did not differ significantly between the control group (G1: 25.64 ± 2.51 U/L) and the fenugreek-only group (G2: 23.43 ± 2.51 U/L; mean difference = 2.21, *p* = 0.372), indicating that fenugreek alone did not perturb hepatic function. Pb exposure (G3) significantly elevated ALT (48.12 ± 2.51 U/L) relative to both G1 and G2 (*p* < 0.0001), consistent with hepatocellular injury.

Co-administration of fenugreek with lead progressively reduced ALT in G4 (33.84 ± 2.51), G5 (38.72 ± 2.51), and G6 (42.89 ± 2.51 U/L) compared with the Pb-only group (G3). Under the pre-specified focused comparisons, this reduction reached significance only at the lowest Pb co-exposure (G4 vs. G3, *p* = 0.002), whereas G5 and G6 did not differ significantly from G3 (*p* = 0.058 and *p* = 0.648). ALT values remained above the control. The protective effect was greatest at the lowest Pb co-exposure (G4) and diminished as the Pb dose increased (G5 and G6), consistent with finite protective capacity at the fixed 200 mg/kg fenugreek dose.

Mean ALT followed a dose-related order, although only the lowest Pb co-exposure (G4) differed significantly from Pb-only after a multiplicity control. ALT was lowest in G4 (Pb 50 mg/kg + fenugreek), followed by G5 (Pb 100 mg/kg) and G6 (Pb 150 mg/kg). Fenugreek partly corrected lead-induced hepatotoxicity in all of the co-treated groups (G4–G6), which had much lower ALT levels than G3. Significant variations in protective responses were seen across the co-treated groups, including G4 vs. G5 (*p* = 0.0008), G4 vs. G6 (*p* < 0.0001), and G5 vs. G6 (*p* = 0.006).

Serum aspartate aminotransferase (AST) levels showed a highly significant variation among the six experimental groups. Because the data exhibited unequal variances, robust statistical analyses were performed using Brown–Forsythe and Welch’s ANOVA tests. The Brown–Forsythe test revealed a significant difference among group means, F (5,29.16) = 126.8, *p* < 0.0001, which was further confirmed by Welch’s ANOVA W, (5,24.29) = 249.5, *p* < 0.0001, indicating a strong treatment effect on serum AST levels (Figure 4b).

Descriptive analysis showed marked differences in AST activity across the groups. The highest AST level was observed in Group 3 (112.2 ± 5.59 U/L), followed by Group 6 (102.9 ± 6.65 U/L), Group 5 (93.02 ± 1.32 U/L), and Group 4 (87.79 ± 1.19 U/L). Lower AST levels were recorded in Group 2 (73.48 ± 6.32 U/L) and Group 1 (71.63 ± 1.65 U/L).

Post hoc analysis using the Games–Howell multiple comparison test showed that Group 3 had significantly higher AST levels than all of the other groups. Under the pre-specified focused comparisons, Group 3 was significantly higher than the control (G1, *p* < 0.0001), and fenugreek significantly lowered AST relative to Pb-only at the lowest Pb co-exposure (G4 vs. G3, *p* = 0.001), whereas G5 and G6 did not differ significantly from G3 (*p* = 0.109 and *p* = 0.370). Comparisons among the co-treatment groups (G4–G6) were treated as exploratory. No significant difference was observed between Group 1 and Group 2 (*p* > 0.05), indicating comparable baseline enzyme levels.

Serum alkaline phosphatase (ALP) levels showed highly significant variation among the six experimental groups. Because of unequal variances, robust statistical analysis was performed using Brown–Forsythe and Welch’s ANOVA tests. The Brown–Forsythe ANOVA confirmed a significant difference among group means (F(5, 16.33) = 189.5, *p* < 0.0001), as did Welch’s ANOVA (W(5, 24.71) = 358.5, *p* < 0.0001), indicating a strong treatment effect on ALP activity (Figure 4c).

Based on statistics, Group 3 had the highest ALP level (98.91 ± 8.88 U/L), followed by Group 6 (83.67 ± 2.52), Group 5 (72.50 ± 1.50), and Group 4 (60.40 ± 3.95 U/L). In contrast, Group 1 (52.70 ± 1.61 U/L) and Group 2 (52.68 ± 1.78 U/L) had the lowest ALP levels, with virtually equal mean values.

Under the pre-specified focused comparisons, Group 3 was markedly elevated versus the control (G1, *p* < 0.0001), and fenugreek significantly lowered ALP relative to Pb-only at the lowest Pb co-exposure (G4 vs. G3, *p* = 0.001); G5 and G6 did not differ significantly from G3 (*p* = 0.064 and *p* = 0.661). No statistically significant difference was observed between Group 1 and Group 2 (*p* > 0.9999), indicating similar baseline ALP activity.

ALT, AST, and ALP were each lower in the fenugreek co-treated groups (G4–G6) than in the Pb-only group (G3), although all three remained above the control levels. (Mechanistic interpretation of this pattern is presented in Section 4).

**Figure 5:**
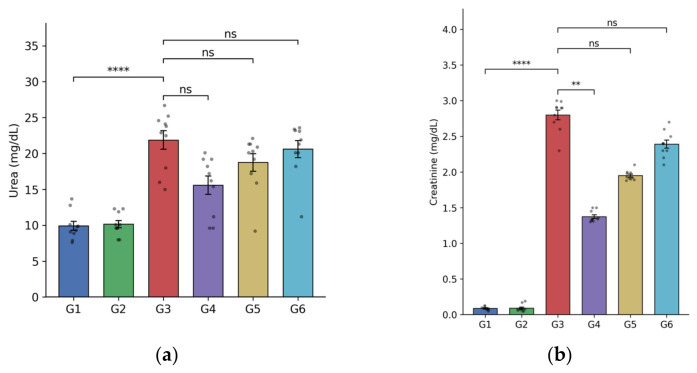
Kidney mouse cohort exam. (**a**) Urea levels (mmol/L): The bar graph showing the mean ± SD for the six groups (G1–G6). Minimum difference between G1 and G2. Under the pre-specified focused comparisons, urea was significantly elevated in Pb-only versus the control (G3 vs. G1, **** *p* < 0.0001). None of the fenugreek co-treatment groups (G4, G5, G6) differed significantly from Pb-only (G3; ns), so significant urea-based renal protection was not established. (**b**) Creatinine (mg/dL) in the six experimental groups. The bar graphs show the mean ± SEM serum creatinine concentrations for Groups 1–6. Group 3 had the highest creatinine levels (2.80 ± 0.22 mg/dL), while Groups 1 and 2 had similar baseline values (~0.09 mg/dL). Statistical analysis followed the data-driven scheme in Section 2.10 (Kruskal–Wallis with Dunn’s test; see Supplementary Table S1). Under the focused comparisons, creatinine was significantly lowered relative to Pb-only at the lowest Pb co-exposure (G4 vs. G3, ** *p* = 0.001); G5 and G6 did not differ significantly from Pb-only (ns). Significance levels: *** *p* < 0.001; **** *p* < 0.0001. Ns, not significant. Per-group animal/sample count is n. The omnibus test and post hoc procedure for each panel are reported in Supplementary Table S1.

Serum urea levels (mmol/L) varied significantly among the six experimental groups (G1–G6) (Figure 5a). The omnibus test and effect size are reported with the focused analysis below and in Supplementary Table S1 (Figure 5a).

Both renal markers were strongly affected by treatment (full statistics in Supplementary Table S1). Urea: Kruskal–Wallis H = 38.4, *p* < 0.0001, ε^2^ = 0.62; Pb-only vs. control (G3 vs. G1) **** (*p* < 0.0001); fenugreek-only vs. control (G2 vs. G1) ns (*p* = 1); and co-treatment vs. Pb-only (Dunn (Holm)) in G4 ns (*p* = 0.052), G5 ns (*p* = 0.5112), and G6 ns (*p* = 1). Creatinine: Kruskal–Wallis H = 55.3, *p* < 0.0001, ε^2^ = 0.93; Pb-only vs. control (G3 vs. G1) **** (*p* < 0.0001); fenugreek-only vs. control (G2 vs. G1) ns (*p* = 0.7978); and co-treatment vs. Pb-only (Dunn (Holm)) in G4 ** (*p* = 0.0012), G5 ns (*p* = 0.0586), and G6 ns (*p* = 0.5811).

The assumption test yielded excellent parametric conditions. The Brown–Forsythe test indicated no variance heterogeneity (*p* = 0.3727), although Bartlett’s test showed modest heteroscedasticity (*p* = 0.0352). The results are physiologically reasonable because of the balanced sample size (n = 10 per group) and significant ANOVA.

Both the control (G1) and fenugreek-treated (G2) groups showed comparable blood urea levels (*p* > 0.9999). Compared with G1 and G2, lead exposure in G3 significantly raised urea levels (21.88 ± 4.06 mmol/L) (*p* < 0.0001), indicating severe renal function and nitrogen metabolism damage from Pb.

Mean serum urea was lower in the fenugreek co-treatment groups than in Pb-only; however, under the pre-specified focused comparisons, G4, G5, or G6 did not differ significantly from G3 (*p* = 0.052, 0.511, and 0.999, respectively). Significant renal protection was, therefore, not established for urea, and the renal-protection conclusion rests on creatinine (below).

Mean urea was lowest in G4 and higher in G5 and G6, but these differences from Pb-only were not statistically significant after multiplicity control. For non-significant results, G3 vs. G5 and G3 vs. G6 exhibited minimal and varied protection across treatment dosages.

Serum creatinine levels varied greatly across the six study groups. Brown–Forsythe and Welch’s ANOVA tests were used for robust statistical analysis due to group variance heterogeneity. The Brown–Forsythe ANOVA showed a significant difference in the group means (F (5, 23.48) = 867.1, *p* < 0.0001), and Welch’s ANOVA (W (5, 23.39) = 2040, *p* < 0.0001) revealed a substantial treatment impact on renal function indicators (Figure 5b).

The statistics revealed clear differences among the groups. The highest serum creatinine level was observed in Group 3 (2.80 ± 0.22 mg/dL), followed by Group 6 (2.39 ± 0.18 mg/dL), Group 5 (1.95 ± 0.07 mg/dL), and Group 4 (1.38 ± 0.08 mg/dL). In contrast, markedly lower creatinine levels were recorded in Group 2 (0.092 ± 0.048 mg/dL) and Group 1 (0.089 ± 0.024 mg/dL), with both groups showing nearly identical baseline values.

Post hoc analysis using the Games–Howell multiple comparison test showed that Group 3 had significantly higher creatinine levels than all other groups. Under the pre-specified focused comparisons, Group 3 was markedly elevated versus the control (G1, *p* < 0.0001), and fenugreek significantly lowered creatinine relative to Pb-only at the lowest Pb co-exposure (G4 vs. G3, *p* = 0.001); G5 and G6 did not differ significantly from G3 (*p* = 0.059 and *p* = 0.581). Group 6 also showed significantly higher creatinine than Groups 4 and 5, and Group 5 was significantly elevated relative to Group 4. No significant difference was observed between Group 1 and Group 2 (*p* > 0.9999), indicating comparable baseline renal function.

Fenugreek co-treatment significantly reduced blood urea and creatinine in G4, G5, and G6 relative to the Pb-only group, indicating partial recovery of renal function. Pb is known to induce nephrotoxicity and accumulation of nitrogenous waste through oxidative damage to renal tubules and a reduction in glomerular filtration rate. Fenugreek flavonoids, saponins, and polyphenols can stabilize nephron membranes and limit lipid peroxidation of renal tissue, supporting filtration efficiency and the corresponding decreases in urea and creatinine. The dose-graded response across G4–G6 indicates that protective capacity at the fixed 200 mg/kg fenugreek dose is incomplete at the highest Pb co-exposure.

### 3.4. Lead Concentration in the Blood

**Figure 6:**
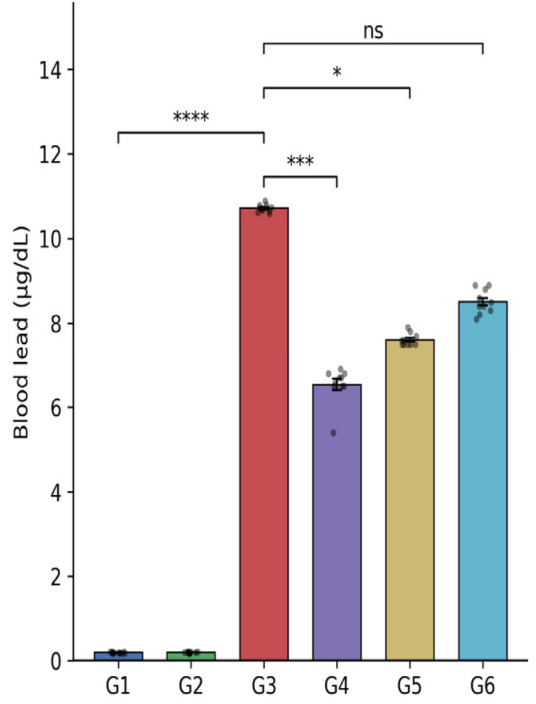
Blood lead levels (µg/dL) after lead exposure and fenugreek therapy in the experimental groups. The bar graphs show the mean ± SEM blood lead levels in the control, Feno200, Pb150, Pb50_Feno200, Pb100_Feno200, and Pb150_Feno200 groups. The control and Feno200 groups had low baseline lead levels (~0.19–0.20 µg/dL), but the Pb150 group had the highest blood lead content (10.72 ± 0.094 µg/dL), indicating effective lead toxicity induction. The fenugreek co-treatment groups (Pb50_Feno200, Pb100_Feno200, and Pb150_Feno200) showed gradually higher lead levels (6.54, 7.61, and 8.51 µg/dL), indicating partial, dose-dependent lead accumulation mitigation. The analysis followed the data-driven scheme in Section 2.10 (Kruskal–Wallis with Dunn’s test; see Supplementary Table S1). Under the focused comparisons, blood lead was significantly lowered relative to Pb-only at the two lower co-exposures (G4 vs. G3, *** *p* < 0.001; G5 vs. G3, * *p* = 0.041) but not in G6 (ns). Significance levels: *** *p* < 0.001; **** *p* < 0.0001. Ns, not significant. Per-group animal count is n. The omnibus test and post-hoc procedure for each panel are reported in Supplementary Table S1.

Due to variance heterogeneity, Brown–Forsythe and Welch’s ANOVA assessed the effects of interventions on blood lead levels. Significant changes in blood lead concentrations were seen among the groups (Brown–Forsythe ANOVA: F (5, 19.30) = 4033, *p* < 0.0001; Welch’s ANOVA: W(5, 23.42) = 28,874, *p* < 0.0001), suggesting a robust treatment impact (Figure 6 and Table 7).

Blood lead confirmed exposure and partial mitigation (full statistics in Supplementary Table S1). Blood lead: Kruskal–Wallis H = 56.0, *p* < 0.0001, ε^2^ = 0.94; Pb-only vs. control (G3 vs. G1) **** (*p* < 0.0001); fenugreek-only vs. control (G2 vs. G1) ns (*p* = 0.7877); and co-treatment vs. Pb-only (Dunn (Holm)) in G4 *** (*p* = 0.0007), G5 * (*p* = 0.0413), G6 ns (*p* = 0.3995).

Statistics varied greatly among the experimental groups. The control group had low baseline lead levels (0.193 ± 0.014 µg/dL), whereas the fenugreek-only group had no change (Feno200: 0.198 ± 0.014 µg/dL), demonstrating that fenugreek alone did not affect physiological lead levels. In contrast, the Pb150 group saw a significant increase in blood lead content (10.72 ± 0.094 µg/dL), indicating effective lead poisoning induction.

Co-treatment with fenugreek resulted in a reduction in blood Pb, partly reflecting the lower administered Pb dose, and that the unique fenugreek effect is best quantified by comparing G6 (Pb 150 + fenugreek) vs. G3 (Pb 150 alone); here, BLL drops from 10.72 to 8.51 μg/dL, a ~21% reduction at the same Pb dose. This is the clean ‘fenugreek effect’.

Post hoc analysis using the Games–Howell test revealed no significant difference between the control and fenugreek-only groups (*p* = 0.965), confirming the absence of an intrinsic effect of fenugreek on basal lead levels. However, all of the lead-exposed groups showed statistically significant increases compared with the control (*p* < 0.0001), confirming successful toxicity induction.

Although all of the lead-exposed groups remained well above the control (*p* < 0.0001), under the pre-specified focused comparisons, fenugreek significantly lowered blood lead relative to Pb-only at the two lower co-exposures (G4 vs. G3, *p* < 0.001; G5 vs. G3, *p* = 0.041), whereas G6 did not differ significantly from G3 (*p* = 0.40), consistent with a graded but dose-limited reduction.

The reduction in blood Pb across all three of the co-treatment groups is consistent with reported metal-chelating and absorption-limiting effects of fenugreek phytochemicals and soluble fiber [6,8], and likely contributes to the parallel improvements in hepatic and renal biomarkers documented above.

### 3.5. Histological Findings

**Figure 7:**
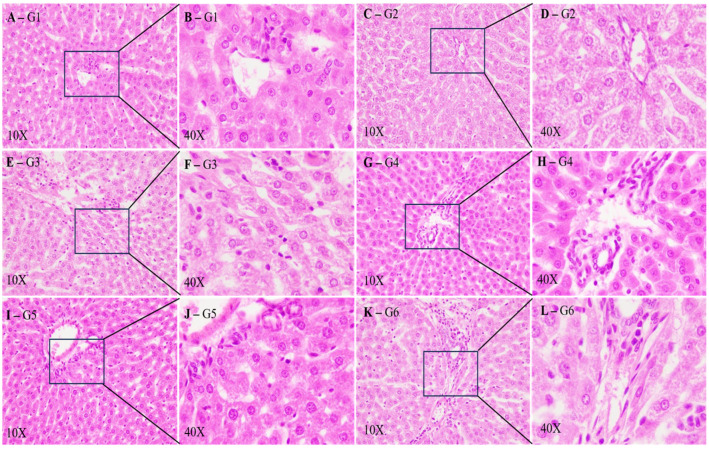
Representative photomicrographs of hematoxylin & eosin (H&E)-stained liver sections across the six experimental groups (G1–G6). For each group, the left panel shows a low-magnification field (10×) with a boxed region, and the right panel shows the corresponding high-magnification view (40×) of that boxed region. Histological injury was assessed by blinded semi-quantitative grading (scale 0–4) for necrosis, inflammation, and cytoplasmic vacuolation; reported values are means of two readings. Hepatic architecture was normal in G1 and G2, whereas G3–G6 displayed necrotic, inflammatory, and vacuolar alterations, with G3 the most severely affected. (**A**,**B**) – G1 (Control): Intact hepatocyte cords, well-preserved nuclei, and normal central veins. Necrosis, inflammation, and vacuolation scores = 1 each. Most hepatocyte nuclei and cytoplasm are free of necrotic foci, with clean sinusoids and no perivascular clustering. (**A**) 10×; (**B**) 40×. (**C**,**D**) – G2 (Fenugreek-treated): Liver cells closely resemble the control. Necrosis, inflammation, and vacuolation scores = 1 each. Most hepatocyte nuclei and cytoplasm show no necrotic foci, sinusoids are clean, perivascular clustering is absent, and inflammation is low. (**C**) 10×; (**D**) 40×. (**E**,**F**) – G3 (Lead-exposed): The most severely affected group. Necrosis, inflammation, and vacuolation scores = 4 each. Marked eosinophilia and loss of cellular architecture indicate cell death; dispersed lymphocytes and other leukocytes with small, dark nuclei represent inflammatory infiltrate; extensive vacuolation with prominent cytoplasmic vacuoles indicates widespread injury. Scattered hepatocellular degeneration, small necrotic foci, cytoplasmic vacuolation, and periportal inflammatory infiltration are evident. (**E**) 10×; (**F**) 40×. (**G**,**H**) – G4: Necrosis = 2, inflammation = 1, vacuolation = 2. A pale central area with reduced cellular detail indicates regional necrosis, surrounded by numerous dark-staining nuclei reflecting inflammation. Most hepatocytes show rounded cytoplasmic gaps, though fewer than in the more severely affected groups. (**G**) 10×; (**H**) 40×. (**I**,**J**) – G5: Mild changes. Necrosis = 2, inflammation = 2, vacuolation = 2. Cytoplasmic eosinophilia and blurred cell borders indicate necrosis; scattered inflammatory cells with dark nuclei are present. These hepatocytes contain fewer cytoplasmic vacuoles than the moderate or severe groups. (**I**) 10×; (**J**) 40×.(**K**,**L**) – G6: Necrosis, inflammation, and vacuolation scores = 3 each. Increased eosinophilia, loss of cellular detail, and ghost-like cell outlines indicate necrosis; several dark-staining nuclei around the necrotic region indicate inflammation. Some hepatocytes show mild cytoplasmic vacuolation. (**K**) 10×; (**L**) 40×.

The control group’s H&E-stained liver slices exhibited intact hepatocyte cords, nuclei, and central veins (Figure 7A,B). The fenugreek-treated liver cells resembled the controls (Figure 7C,D). Figure 7E,F show scattered hepatocellular degeneration, minor necrotic foci, cytoplasmic vacuolation, and periportal inflammatory infiltration in Pb-exposed animals. Fenugreek with lead decreased hepatic cord inflammation, necrosis, and disruption.

The following group findings were obtained using blinded semi-quantitative histological grading (0–4). All findings are two-value means. Group G1 has low necrosis, inflammation, and vacuolation (all scores = 1). Most hepatocyte nuclei and cytoplasm lack necrotic foci, with low inflammation and clean sinusoids without perivascular clustering. Group G2 also has scores of 1 for necrosis, inflammation, and vacuolation. Most hepatocyte nuclei and cytoplasm have no necrotic foci, clean sinusoids, no perivascular clustering, and low inflammation.

Group G3 has scores of 4 for necrosis, inflammation, and vacuolation. Eosinophilia and cell architecture loss indicate death. Dispersed lymphocytes or other leukocytes with tiny, black nuclei are inflammatory cells. Many cells have extensive vacuolation and distinctive cytoplasmic vacuoles, suggesting injury.

Necrosis, inflammation, and vacuolation scores are 2, 1, and 2 in Group G4. A pale center with reduced cellular detail indicates regional necrosis, whereas many dark-staining nuclei around it indicate inflammation. Most hepatocytes have rounded cytoplasmic gaps, although less than other groupings.

Group G5 has minor necrosis, inflammation, and vacuolation scores of 2. Cytoplasmic eosinophilia and indistinct cell borders indicate necrosis. Dispersed inflammatory cells have black nuclei. These hepatocytes have fewer cytoplasmic vacuoles than those with moderate or severe vacuolation.

Finally, Group G6 has scores of 3 for necrosis, inflammation, and vacuolation. Necrosis is indicated by increased eosinophilia, cellular detail loss, and ghost-like cell outlines. Several dark-staining nuclei around the necrotic region indicate inflammation. Some hepatocytes have minor cytoplasmic vacuolation.

The control groups (G1 and G2) have normal liver architecture with few pathological findings, while experimental groups (G3–G6) show necrotic, inflammatory, and vacuolar alterations, with G3 being the most seriously impacted. Agreement between the two blinded observers was substantial (unweighted Cohen’s κ = 0.69, 95% CI 0.38–0.92; quadratic-weighted κ = 0.91, 95% CI 0.73–0.98).

### 3.6. Gene Expression Analysis

In order to clarify the molecular basis of Pb toxicity and fenugreek’s protective effects, the relative mRNA expression levels of oxidative stress, inflammatory, and metabolic markers (Nrf2, TNF-α, IL-6, SOD-3, SCD-1, and HO-1) were quantified.

### 3.7. Differential Expression of Oxidative Stress-Related Genes

#### 3.7.1. Nrf2

**Figure 8:**
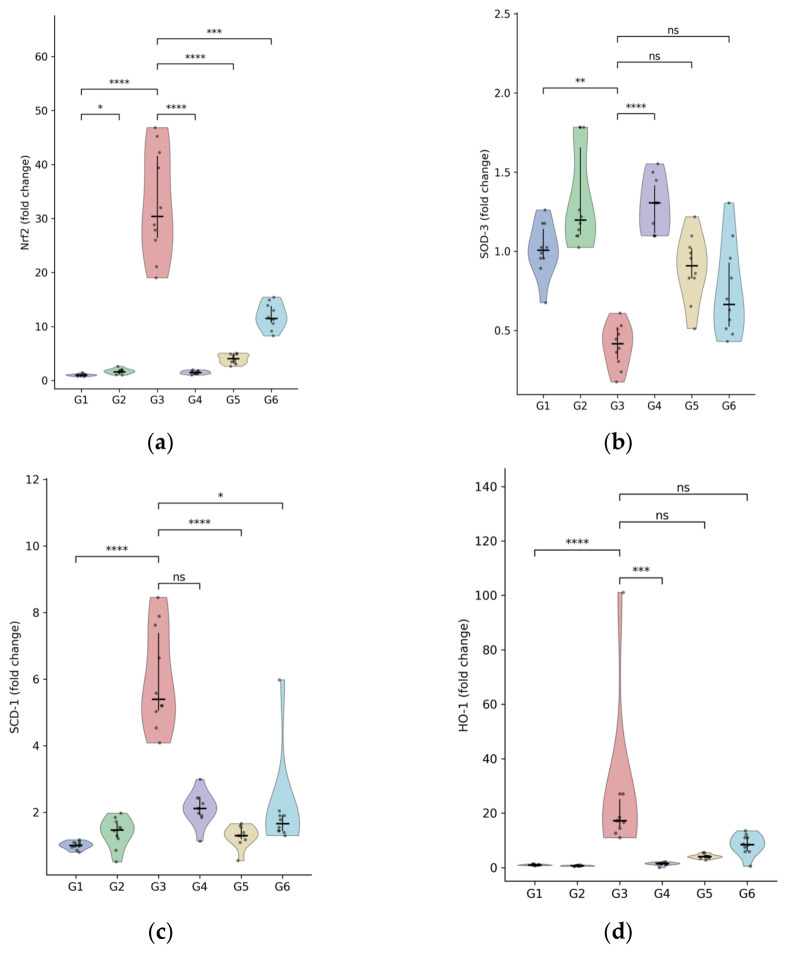
The groups (G1–G6) express oxidative stress genes differently. (**a**) The Nrf2 gene expression fold change in the six experimental groups. The median and interquartile range of Nrf2 fold change values in Groups 1–6. The violin plots are dashed lines. G1 (control) exhibited baseline Nrf2 expression (1.016 ± 0.205), whereas G2 showed a little increase (1.691 ± 0.484). Lead-induced oxidative stress was associated with a large ~33-fold increase in G3 (32.86 ± 9.989), indicating compensatory Nrf2 pathway action. Fenugreek co-administration reduced induction dose-dependently, indicating partial redox equilibrium restoration. G4 (1.504 ± 0.314) returned to near-baseline levels, but G5 (4.136 ± 0.812) and G6 (11.97 ± 2.358) showed intermediate expression levels. Brown–Forsythe and Welch’s ANOVA showed significant group differences (*p* < 0.0001). Games–Howell multiple comparison tests were employed for post hoc pairwise comparisons. Significance levels: ** *p* < 0.01; **** *p* < 0.0001. Ns, not significant. Animal count per category is n. (**b**) The fold change in SOD-3 gene expression in the six experimental groups. Groups 1–6 (G1–G6) violin plots depict SOD-3 fold change distributions with median and interquartile range dotted lines (n = 10 per group). G1 (control) and G2 (fenugreek-only) showed similar antioxidant enzyme expression levels (1.014 ± 0.166 and 1.336 ± 0.314). SOD-3 expression was significantly reduced in G3 (0.404 ± 0.138), indicating lead-induced antioxidant defense system impairment. Fenugreek co-administration restored SOD-3 expression dose-dependently, with G4 (1.291 ± 0.170) recovering near-normal, and G5 (0.897 ± 0.209) and G6 (0.752 ± 0.292) recovering somewhat compared with Pb. The omnibus test and post hoc procedure for SOD-3 followed the data-driven scheme in Section 2.10 (Kruskal–Wallis with Dunn’s test; see Supplementary Table S1). Under the focused comparisons, SOD-3 was restored significantly only at the lowest Pb co-exposure (G4 vs. G3, **** *p* < 0.0001); G5 and G6 did not differ significantly from Pb-only (ns). Significance levels: * *p* < 0.05; ** *p* < 0.01; *** *p* < 0.001; **** *p* < 0.0001. Ns, not significant. (**c**) The fold change in SCD-1 gene expression in the six experimental groups. The dashed lines represent the SCD-1 fold change median and interquartile range in Groups 1–6’s violin plots. G1 (control) had baseline SCD-1 expression (1.006 ± 0.115), whereas G2 (1.389 ± 0.445) and G5 (1.291 ± 0.319) met control levels. G3 exhibited the greatest SCD-1 overexpression (6.024 ± 1.522), indicating lead-induced lipid metabolic disruption. G4 exhibited a moderate but substantial increase over baseline, whereas G6 (2.174 ± 1.409) showed similar intermediate expression. Fenugreek’s dose-dependent decrease in lead-induced SCD-1 overexpression supports hepatometabolic modulation of lipid homeostasis during heavy-metal poisoning. The results from the Brown–Forsythe and Welch’s ANOVA showed significant group differences (*p* < 0.0001). Post hoc pairwise comparisons used Games–Howell multiple comparison tests. Significance levels: **** *p* < 0.0001. Ns, not significant. The number of animals per category is n. (**d**) HO-1 gene expression (fold change) in the six experimental groups. The dotted lines in violin plots show the median and interquartile range of the HO-1 fold change values in Groups 1–6 (G1–G6) (n = 10). Although G1 (control) had baseline HO-1 expression (1.020 ± 0.205), G2 exhibited a minor reduction (0.708 ± 0.160) compared with the control. The highest HO-1 overexpression (19.02 ± 6.237) was seen in G3, showing activated oxidative stress response pathways following lead exposure. G4 (1.420 ± 0.585) remained around baseline, but G5 (4.156 ± 0.820) and G6 (8.457 ± 3.800) demonstrated considerable dose-dependent HO-1 activation under different treatment conditions. Fenugreek co-administration inhibited lead-induced HO-1 overexpression, partially restoring redox equilibrium and oxidative stress signaling. Statistical analysis using Brown–Forsythe and Welch’s ANOVA revealed significant group differences (*p* < 0.0001). Games–Howell multiple comparison tests were employed for post hoc pairwise comparisons. Significant levels: * *p* < 0.05; **** *p* < 0.0001. Ns, not significant. The omnibus test and post hoc procedure for each panel are reported in Supplementary Table S1.

Due to group differences, Brown–Forsythe and Welch’s ANOVA were used to compare Nrf2 expression after different treatments. Significant changes in Nrf2 levels were seen among the groups (Brown–Forsythe ANOVA: F (5, 10.20) = 87.40, *p* < 0.0001; Welch’s ANOVA: W (5, 23.69) = 81.51, *p* < 0.0001), showing treatment-dependent regulation (Figure 8a).

The oxidative-stress and lipid-metabolism genes each showed a strong treatment effect (full statistics in Supplementary Table S1). Nrf2: Welch’s ANOVA F(5,23.7) = 77.0, *p* < 0.0001, η^2^ = 0.89; Pb-only vs. control (G3 vs. G1) **** (*p* < 0.0001); fenugreek-only vs. control (G2 vs. G1) * (*p* = 0.0147); and co-treatment vs. Pb-only (Games–Howell) in G4 **** (*p* < 0.0001), G5 **** (*p* < 0.0001), G6 *** (*p* = 0.0008). SOD-3: Kruskal–Wallis H = 40.8, *p* < 0.0001, ε^2^ = 0.66; Pb-only vs. control (G3 vs. G1) ** (*p* = 0.0063); fenugreek-only vs. control (G2 vs. G1) ns (*p* = 0.2564); and co-treatment vs. Pb-only (Dunn (Holm)) in G4 **** (*p* < 0.0001), G5 ns (*p* = 0.0696), G6 ns (*p* = 0.2564). SCD-1: Kruskal–Wallis H = 43.6, *p* < 0.0001, ε^2^ = 0.71; Pb-only vs. control (G3 vs. G1) **** (*p* < 0.0001); fenugreek-only vs. control (G2 vs. G1) ns (*p* = 0.1766); and co-treatment vs. Pb-only (Dunn (Holm)) in G4 ns (*p* = 0.1766), G5 **** (*p* < 0.0001), G6 * (*p* = 0.0281). HO-1: Kruskal–Wallis H = 48.7, *p* < 0.0001, ε^2^ = 0.81; Pb-only vs. control (G3 vs. G1) **** (*p* < 0.0001); fenugreek-only vs. control (G2 vs. G1) ns (*p* = 0.5471); and co-treatment vs. Pb-only (Dunn (Holm)) in G4 *** (*p* = 0.0004), G5 ns (*p* = 0.0701), G6 ns (*p* = 0.2956).

Statistical analysis showed distinct group-wise variation in Nrf2 expression. The control group (G1) exhibited baseline expression (1.016 ± 0.205), which slightly increased in G2 (1.691 ± 0.484). A pronounced elevation was observed in G3 (32.86 ± 9.989), indicating a strong induction of Nrf2 expression under this condition. In contrast, G4 (1.504 ± 0.314) remained comparable to baseline levels, while moderate increases were observed in G5 (4.136 ± 0.812) and G6 (11.97 ± 2.358), suggesting partial activation of the Nrf2 pathway depending on treatment intensity.

Most groups showed significant differences after Games–Howell post hoc analysis. Nrf2 expression was considerably changed in all of the groups except G4, where it was just slightly different (*p* = 0.0091) from the control (G1). G2 had a moderate but significant increase compared with G1 (*p* = 0.0150), whereas G3 showed a very significant upregulation (*p* < 0.0001). Both G5 and G6 showed substantial rises compared with the control (*p* < 0.0001), with G6 having a greater impact. G2 and G4 had similar baseline responses (*p* = 0.9022).

G3 was the greatest inducer of Nrf2 expression, with significant differences from other groups (*p* < 0.0001 in most comparisons). Additionally, G5 and G6 showed substantial differences (*p* < 0.0001), demonstrating dose-dependent regulation among treatment groups.

The Pb-only group showed a significant increase in Nrf2 (~33-fold over control), likely due to ROS buildup and compensatory antioxidant response activation. Fenugreek co-administration reduced this induction in a Pb-dose-dependent way (G4 < G5 < G6), with Nrf2 expression around baseline in G4. This trend supports the idea that fenugreek decreases upstream oxidative stress, lowering the requirement for compensatory Nrf2 activation, rather than directly suppressing Nrf2.

#### 3.7.2. SOD-3

SOD-3 gene expression varied significantly among the six experimental groups (G1–G6; Figure 8b). The omnibus test and effect size for this endpoint are reported in the focused analysis below and in Supplementary Table S1.

Since Brown–Forsythe (*p* = 0.3210) and Bartlett’s (*p* = 0.0947) were non-significant, assumption testing validated data homogeneity. This indicates that parametric analysis works for group comparisons.

Both the control (G1: 1.014 ± 0.166) and fenugreek-treated (G2: 1.336 ± 0.314) groups had higher antioxidant enzyme levels. Lead-induced oxidative stress in G3 decreased SOD-3 expression (0.404 ± 0.138) relative to the control (G3 vs. G1, *p* = 0.002). Under the focused comparisons, restoration by fenugreek was significant only at the lowest Pb co-exposure (G4 vs. G3, *p* < 0.0001), with G5 and G6 not differing significantly from G3. This reduces antioxidant protection.

Co-administration of fenugreek with Pb significantly restored SOD-3 expression in a dose-graded manner. G4 (1.291 ± 0.170) recovered to near-control values, while G5 (0.897 ± 0.209) and G6 (0.752 ± 0.292) showed significant but partial recovery relative to the Pb-only group (*p* < 0.001).

Tukey’s post hoc analysis showed that Pb exposure significantly inhibited SOD-3 expression, although fenugreek co-treatment partially corrected this suppression depending on Pb dosage.

The significant downregulation of SOD-3 in Pb-exposed mice suggests oxidative stress-mediated antioxidant defense deficiency. At lower Pb co-exposure (G4), the fenugreek co-treatment restored SOD-3 expression to control levels, showing it can boost cellular antioxidant defenses and reduce oxidative damage.

#### 3.7.3. SCD-1

Due to group differences, Brown–Forsythe and Welch’s ANOVA were used to compare SCD-1 expression after different treatments. Both analyses showed significant changes among groups (Brown–Forsythe ANOVA: F (5, 22.47) = 43.16, *p* < 0.0001; Welch’s ANOVA: W(5, 22.64) = 30.12, *p* < 0.0001), showing treatment-dependent SCD-1 expression modulation (Figure 8c).

The experimental groups had different SCD-1 levels, according to statistical analysis. The control group (G1) showed baseline expression (1.006 ± 0.115) and remained stable in G2 (1.389 ± 0.445), showing no significant change after treatment. SCD-1 expression increased significantly in G3 (6.024 ± 1.522), indicating robust gene induction.

In contrast, moderate expression levels were observed in G4 (2.119 ± 0.483) and G6 (2.174 ± 1.409), while G5 (1.291 ± 0.319) remained close to baseline levels. Overall, G3 represented the highest SCD-1 expression among all the groups.

Post hoc analysis using the Games–Howell test indicated no significant difference between G1 and G2 (*p* = 0.1730), G1 and G5 (*p* = 0.1593), or G1 and G6 (*p* = 0.1871). These treatments had no influence on the control. However, G3 had significantly higher SCD-1 expression than other groups (*p* < 0.0001–0.0002), indicating substantial upregulation. G4 was elevated relative to the control (*p* = 0.0003), but lower than G3.

Under the pre-specified focused comparisons, lead-induced SCD-1 overexpression (G3) was significantly reduced by fenugreek at higher Pb co-exposures (G5 vs. G3, *p* < 0.0001; G6 vs. G3, *p* = 0.028), whereas G4 did not differ significantly from G3 (*p* = 0.18). The response was, therefore, irregular rather than dose-graded. This nonetheless suggests fenugreek may help restore lipid–metabolic balance under heavy-metal stress.

#### 3.7.4. HO-1

The effect of different treatments on HO-1 expression was evaluated using Brown–Forsythe and Welch’s ANOVA due to the heterogeneity of variances among the groups. Both analyses showed highly significant differences among the groups (Brown–Forsythe ANOVA: F*(5, 15.48) = 55.66, *p* < 0.0001; Welch’s ANOVA: W (5, 23.52) = 55.05, *p* < 0.0001), indicating a strong treatment-dependent modulation of HO-1 expression (Figure 8d).

Statistical analysis showed significant HO-1 expression variance among the experimental groups (n = 10). The control group (G1) had the baseline expression (1.020 ± 0.205), with values ranging from 0.740 to 1.380. A small drop in G2 (0.708 ± 0.160; range: 0.490–0.970) indicates moderate suppression compared with the control.

G3 showed a significant increase in HO-1 expression (19.02 ± 6.237), ranging from 11.00 to 28.10, indicating a robust oxidative stress response mechanism. In contrast, G4 levels remained near baseline (1.420 ± 0.585; range: 0.020–2.160), indicating little activation.

Moderate increases were observed in G5 (4.156 ± 0.820; range: 2.850–5.500) and G6 (8.457 ± 3.800; range: 0.520–13.55), indicating dose-dependent upregulation of HO-1 expression under different treatment conditions.

The median and interquartile range further supported these trends, with G3 showing the highest median value (17.27), while G1 and G2 remained low (0.940 and 0.690, respectively), confirming consistency with mean-based observations.

Post hoc analysis revealed significant differences among most of the groups. Compared with the control (G1), HO-1 expression was significantly increased in G2 (*p* = 0.0152), G5 (*p* < 0.0001), and G6 (*p* = 0.0015), while G4 showed no significant difference (*p* = 0.3786), indicating a negligible effect in this group.

Under the pre-specified focused comparisons, HO-1 was strongly induced by Pb (G3 vs. control, *p* < 0.0001), and fenugreek significantly attenuated this overexpression only at the lowest Pb co-exposure (G4 vs. G3, *p* < 0.001); G5 and G6 did not differ significantly from G3 (*p* = 0.070 and *p* = 0.296). Because a single high-leverage value widened the spread of the Pb-only group, non-parametric testing was applied. Pb exposure thus produced marked HO-1 upregulation consistent with activation of the antioxidant response, with significant fenugreek attenuation limited to the lowest Pb co-exposure.

### 3.8. Differential Expression of Inflammation-Related Genes

#### 3.8.1. TNF-α

**Figure 9:**
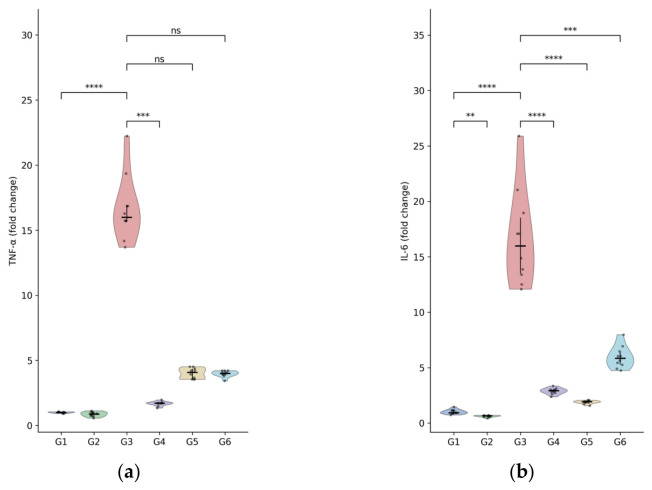
Differences in G1–G6 inflammatory gene expression among the groups. (**a**) TNF-α fold-change expression in the 6 groups with similar baselines in G1 and G2 (ns). TNF-α levels in G3 were significantly greater than both of the controls (**** *p* < 0.0001), demonstrating Pb-induced inflammation. Under the pre-specified focused comparisons, fenugreek significantly reduced TNF-α relative to Pb-only at the lowest Pb co-exposure (G4 vs. G3, *** *p* < 0.001); G5 and G6 did not differ significantly from Pb-only (ns) and remained above the control. (**b**) The violin plots show IL-6 expression fluctuations in the experimental groups G1 and G2, with comparable baseline expression (ns). G3 substantially raised IL-6 levels relative to the controls (**** *p* < 0.0001), indicating a pro-inflammatory effect. Protective normalization occurred between G4 and G5. G6 had higher IL-6 levels than both of the controls (**** *p* < 0.0001), but much lower than G3 (**** *p* < 0.0001). Pairwise analysis indicated that G4 and G5 were significantly lower than G6 (** *p* = 0.0056; *** *p* = 0.0001), suggesting fenugreek’s protective effect. At lower Pb levels (Pb100), fenugreek supplements completely stopped the rise in IL-6, but at higher levels (Pb150), they somewhat decreased the inflammatory response. The omnibus test and post hoc procedure for each panel are reported in Supplementary Table S1.

TNF-α expression differed significantly among the six experimental groups (Figure 9a; omnibus test and effect size reported with the analysis below and in Supplementary Table S1), indicating strong treatment-dependent modulation of the inflammatory response. The Pb-only group (G3) had significantly elevated TNF-α relative to controls, consistent with a marked pro-inflammatory effect of Pb. Under the pre-specified focused comparisons, fenugreek significantly reduced TNF-α relative to Pb-only at the lowest Pb co-exposure (G4 vs. G3, *p* < 0.001), whereas G5 and G6 did not differ significantly from G3.

Both inflammatory genes showed a strong treatment effect with fenugreek attenuation (full statistics in Supplementary Table S1). TNF-α: Kruskal–Wallis H = 54.6, *p* < 0.0001, ε^2^ = 0.92; Pb-only vs. control (G3 vs. G1) **** (*p* < 0.0001); fenugreek-only vs. control (G2 vs. G1) ns (*p* = 0.5555); and co-treatment vs. Pb-only (Dunn (Holm)) in G4 *** (*p* = 0.0008), G5 ns (*p* = 0.1907), and G6 ns (*p* = 0.1907). IL-6: Welch’s ANOVA F(5,23.6) = 250.8, *p* < 0.0001, η^2^ = 0.91; Pb-only vs. control (G3 vs. G1) **** (*p* < 0.0001); fenugreek-only vs. control (G2 vs. G1) ** (*p* = 0.0012); and co-treatment vs. Pb-only (Games–Howell) in G4 **** (*p* < 0.0001), G5 **** (*p* < 0.0001), and G6 *** (*p* = 0.0002).

Under the focused comparisons, fenugreek significantly reduced TNF-α expression relative to Pb-only at the lowest Pb co-exposure (G4 vs. G3, *p* < 0.001); reductions in G5 and G6 did not reach significance after multiplicity control, although co-treated groups trended towards the baseline inflammatory state.

Pb exposure elicited a significant inflammatory response, evidenced by the marked rise in hepatic TNF-α expression in G3. Co-administration of fenugreek attenuated this increase relative to Pb-only exposure, reaching statistical significance at the lowest Pb co-exposure (G4); attenuation at the higher Pb doses did not reach significance after multiplicity control. These results indicate that fenugreek seed powder mitigates Pb-induced TNF-α upregulation in vivo.

#### 3.8.2. IL-6

IL-6 expression differed significantly among the six experimental groups (Figure 9b; the omnibus test and effect size are reported in the analysis below and in Supplementary Table S1), indicating strong treatment-dependent modulation of inflammatory status. The Pb-only group (G3) had significantly higher IL-6 levels than the control (G1), confirming a strong inflammatory response to Pb exposure. Under the focused comparisons, fenugreek significantly decreased IL-6 relative to Pb-only at all three co-exposures (G4, G5, and G6 vs. G3; *p* < 0.001 for each).

Under the focused comparisons, IL-6 was significantly decreased relative to Pb-only (G3) in all of the fenugreek co-treated groups (G4, G5, and G6). Although the co-treated groups remained slightly above the control, a clear and significant reduction relative to Pb-only was detected across all three doses.

Hepatic IL-6 expression was significantly elevated following Pb exposure, consistent with robust pro-inflammatory signaling. Co-administration of fenugreek reduced IL-6 expression in G4, G5, and G6 relative to the Pb-only group, indicating anti-inflammatory activity, with partial restoration of inflammatory homeostasis that diminished as Pb co-exposure increased.

Fenugreek seed likely attenuates lead-induced inflammation by downregulating IL-6 expression, thereby suppressing cytokine-mediated inflammatory damage and contributing to recovery of immune homeostasis in toxic conditions.

## 4. Discussion

In the present study, lead exposure (G3, Pb 150 mg/kg) significantly reduced body-weight gain, food intake, and water consumption relative to control. Across the three Pb co-treatment groups (G4–G6), the magnitude of these decrements increased with an increasing Pb dose. Pb-induced anorexia and reduced growth are well documented in rodent toxicology and have been attributed to oxidative-stress-mediated disruption of hypothalamic appetite regulation, neuroendocrine signaling, and gastrointestinal function. Co-administration of fenugreek seed powder partially restored these parameters across all three of the Pb co-exposure groups. Even with a constant dose, fenugreek increased food and water intake and partially restored body weight. Fenugreek saponins, flavonoids, and galactomannans have been reported to mitigate toxin-induced anorexia through antioxidant, anti-inflammatory, and prebiotic mechanisms [29].

Because whole, unfractionated seed powder was administered and the 32 constituents were identified only tentatively, the following structure–activity relationships are offered as hypotheses to guide future bioactivity-guided fractionation, rather than as demonstrated mechanisms, since no individual constituent was isolated or tested in the present study. With that caveat, the dominant compound classes detected by LC–DAD–ESI–MS/MS have bioactivities reported in the literature that are directionally consistent with the observed protection. (i) Vicenin-type apigenin C-glycosides (vicenin-1, -2, and -3), which together accounted for ~33% of the integrated peak area, have been reported to possess antioxidant and anti-inflammatory activity. Importantly, a reduction in the upstream oxidative and inflammatory burden of Pb would be expected to lower the compensatory induction of Nrf2 and HO-1 and to reduce NF-κB-driven TNF-α/IL-6—consistent with the lower (rather than further-elevated) expression of these genes in G4–G6, and avoiding the internal contradiction of invoking direct Nrf2 activation while observing reduced Nrf2 expression. (ii) Luteolin C-glycoside derivatives (the most abundant class, ~15.9%), whose ortho-dihydroxyl B-ring supports radical scavenging, may contribute to the partial recovery of SOD-3 observed at the lowest Pb co-exposure (G4). (iii) Acylated polyphenols carrying galloyl, feruloyl, and malonyl groups provide multiple hydroxyl donors capable of coordinating divalent metals in vitro and could, together with the soluble galactomannan fiber fraction, contribute to the modest reduction in blood Pb (~21% at the matched 150 mg/kg Pb dose, G6 vs. G3); however, direct evidence, such as in vitro Pb-binding assays and measurement of fecal, urinary, and tissue Pb, was not obtained here and is required before a chelation/absorption-limiting mechanism can be claimed. (iv) Kaempferol 7-O-rhamnosyl-glucoside and dihydrogallic acid derivatives may add further antioxidant and anti-inflammatory activity. Overall, the phytochemical profile offers a chemically plausible, but not yet experimentally validated, rationale for the multi-axis effects observed.

Pb altered biochemical homeostasis in a dose-dependent manner, producing oxidative stress and functional deterioration. Pb-induced ROS, antioxidant-enzyme inhibition, and mitochondrial dysfunction underpin systemic toxicity, consistent with prior reports on Pb-induced oxidative stress, suppression of endogenous antioxidant defenses, and organ damage. By binding sulfhydryl groups on enzymes and structural proteins, Pb impairs redox-sensitive metabolic activity and promotes lipid peroxidation. Our findings are consistent with prior reports that Pb exposure lowers SOD, CAT, and GPx activity and increases oxidative-stress and inflammatory mediators [30].

Blood Pb concentrations rose in proportion to Pb dose. Pb-exposed animals (G3) showed the largest systemic burden, and fenugreek co-treatment reduced this burden, reaching significance at the two lower co-exposures (G4 and G5 vs. G3), consistent with partial detoxification or reduced bioavailability. Pb is readily absorbed, circulates in blood, and accumulates in soft tissues and bone, creating a long-term internal reservoir that drives oxidative stress, inflammation, and multi-organ injury [4]. The reduction in blood Pb observed across G4–G6 at a fixed fenugreek dose is consistent with reduced intestinal absorption, antioxidant activity, and the metal-chelating capacity of fenugreek phytochemicals. In G3, Pb caused marked hepatic architectural degradation, hepatocellular degeneration, vacuolation, inflammatory infiltration, and necrotic foci, reflecting Pb’s oxidative, membrane-damaging, and Kupffer-cell-activating effects.

ROS-mediated lipid peroxidation and cytokine activation produce sinusoidal dilatation, hepatocyte death, and inflammatory infiltration in Pb-exposed mice [31]. Fenugreek supplementation has been shown to enhance antioxidant defenses, reduce inflammation, and preserve hepatic architecture under toxic stress [7,32]. In our co-treatment groups (G4–G6), histopathological injury was reduced relative to Pb-only exposure (G3), with the greatest preservation at the lowest Pb co-exposure (G4). The pattern across G4–G6 indicates that fenugreek partially stabilizes hepatocellular membranes and limits Pb-induced histological injury, most likely through its combined antioxidant and anti-inflammatory activities.

Pb exposure produced a complex pattern of antioxidant–gene dysregulation: induction of Nrf2 and HO-1, suppression of SOD-3, and upregulation of SCD-1, consistent with an oxidative-stress/cellular-defense imbalance. Pb-induced ROS activate the Nrf2/HO-1 axis as a compensatory cytoprotective response, but ROS production and mitochondrial dysfunction render this response inadequate at high toxic loads [13]. The pronounced SOD-3 suppression in G3 indicates enzymatic-antioxidant failure, while SCD-1 upregulation reflects Pb-induced lipid-metabolic reprogramming. Fenugreek co-treatment attenuated Nrf2/HO-1 over-activation, restored SOD-3, and normalized SCD-1, consistent with reduction in the upstream oxidative stimulus rather than direct inhibition of these transcription factors. Our LC–DAD–ESI–MS/MS profile provides a defined polyphenolic basis for these effects: vicenin-1, -2, and -3 collectively accounted for ~33% of the extract, and vicenin-2 has been shown to activate Nrf2/ARE transcription and suppress NF-κB-mediated cytokine release, directly rationalizing the attenuation of Nrf2 compensatory induction and the reduction in TNF-α and IL-6 in co-treated groups. Luteolin dihydrogalloyl-glucosyl-pentosyl derivatives (15.90%, the most abundant compound class) and kaempferol glycosides (8.71%) contribute further radical-scavenging and metal-coordinating activity, and the numerous acylated flavonoid glycosides bearing galloyl, feruloyl, and malonyl substituents possess additional hydroxyl groups that enhance both radical scavenging and divalent-metal coordination. This polyphenolic chelation capacity provides a mechanistic basis for the dose-graded reductions in blood Pb observed across G4–G6 (*p* < 0.0001 vs. G3). Collectively, the LC–MS/MS data identify vicenin-type apigenin C-glycosides and luteolin derivatives as priority targets for bioactivity-guided fractionation.

Pb exposure also triggered a robust inflammatory response, with significant induction of TNF-α and IL-6, consistent with ROS-driven activation of NF-κB signaling and reported increases in pro-inflammatory cytokines under Pb toxicity [33]. Pb also disrupts mitochondrial integrity and redox equilibrium, sustaining chronic inflammatory transcription [34]. Fenugreek co-treatment significantly reduced TNF-α and IL-6 expression in all Pb co-exposure groups, consistent with antioxidant-mediated dampening of NF-κB activity. The dose-graded recovery across G4–G6—with the strongest attenuation at the lowest Pb co-exposure—reinforces the interpretation that fenugreek’s anti-inflammatory action operates downstream of its antioxidant activity.

In summary, the gene-expression data reveal four convergent Pb-induced perturbations: (i) compensatory induction of Nrf2 and HO-1, (ii) suppression of SOD-3, (iii) dysregulation of SCD-1-dependent lipid metabolism, and (iv) upregulation of TNF-α and IL-6. Fenugreek seed powder reversed these alterations most consistently at the lowest Pb co-exposure, with protection diminishing at higher Pb doses for several endpoints, supporting a coordinated but dose-limited mechanism of redox stabilization and anti-inflammatory regulation. Holding the fenugreek dose constant while varying Pb intensity allowed direct evaluation of protective capacity at a fixed phytochemical dose. The present analysis integrates physiological (body weight and intake), serum-biochemical (ALT, AST, ALP, urea, and creatinine), histological (H&E), and molecular (Nrf2, HO-1, SOD-3, SCD-1, TNF-α, and IL-6) endpoints within the same animals, supporting the interpretation that fenugreek acts as both a systemic antioxidant and a modulator of Pb toxicity, simultaneously attenuating injury pathways and lowering systemic Pb burden.

Several limitations of the present work should be acknowledged. First, the experimental design includes only one Pb-only group (G3, 150 mg/kg). This study, therefore, does not directly establish a dose-response of Pb toxicity, but rather characterizes the protective capacity of a fixed fenugreek dose (200 mg/kg) against three escalating doses of Pb co-exposure. Additional Pb-only dose groups would strengthen any claims about Pb-induced dose–response and are recommended for follow-up. Critically, because Pb-only groups at 50 and 100 mg/kg were not included, the apparently dose-graded protection across G4–G6 cannot be formally distinguished from the progressively lower Pb burden delivered to those groups; the most rigorous within-study estimate of a fenugreek-specific effect is therefore the comparison at matched Pb dose (G6 vs. G3, both 150 mg/kg), where co-treatment produced partial attenuation of biochemical, histological, and molecular injury together with an ~21% reduction in blood Pb. For this reason, references to “dose-graded protection” throughout the manuscript should be read as descriptive of the co-exposure series rather than as evidence of a fenugreek dose–response, and the central protective claim rests primarily on the matched-dose comparison and on the fenugreek-only control. In addition, direct biochemical markers of oxidative stress—such as malondialdehyde, reduced glutathione, and catalase activity—were not measured; this limits direct mechanistic support for the proposed antioxidant action, and their inclusion is recommended in future work. Second, the molecular findings are limited to mRNA-level expression data; protein-level validation by Western blot or ELISA and direct measurement of antioxidant enzyme activities (SOD, CAT, GPx) would substantiate the transcriptional findings. Third, although the present LC–DAD–ESI–MS/MS characterization has identified 32 constituents and confirmed dominance of vicenin-type apigenin C-glycosides and luteolin derivatives, whole seed powder was used in vivo, and the specific protective constituent(s) cannot be determined from these data alone; bioactivity-guided fractionation of specific fenugreek fractions (flavonoid-rich, saponin-rich, galactomannan-rich) is recommended to resolve this. Fourth, only male mice were studied, so the generalizability of the findings to females remains to be tested. Fifth, fenugreek’s effects on Pb absorption, distribution, and excretion should be addressed in dedicated pharmacokinetic studies. Sixth, LC–DAD–ESI–MS/MS characterization of the seed batch used in this study has been performed and is reported herein; while this resolves the previously noted limitation, quantitative profiling of specific marker compounds (e.g., trigonelline by HPLC-UV, diosgenin by GC–MS) and bioactivity-guided fractionation to identify the specific protective constituent(s) remain priorities for future work. Seventh, the histopathological analysis in the present study was confined to liver tissue; renal histology was not performed, and the kidneys from the study cohort are no longer available for retrospective sectioning. Renal function was, however, assessed indirectly through serum urea and creatinine and through Pb accumulation in blood, all of which showed clear Pb-induced changes consistent with nephrotoxicity, which were attenuated by fenugreek co-treatment. Inclusion of renal histopathology alongside hepatic histopathology, and of specific renal injury markers at the transcriptional level (e.g., KIM-1 and NGAL), is recommended for follow-up studies to provide a more comprehensive view of fenugreek’s hepatorenal protective effect. Finally, although the present study included a fenugreek-only control (G2), it did not include a reference pharmacological chelator (e.g., DMSA or D-penicillamine) as a positive control. Comparison with a clinically used chelator would strengthen translational interpretation and is suggested for future work.

## 5. Conclusions

In summary, lead exposure in male albino mice produced a multi-organ toxicity profile characterized by reduced growth performance, hepatorenal biomarker elevation, systemic Pb accumulation, hepatic histopathological injury, and dysregulation of oxidative stress, lipid metabolism, and inflammatory gene expression. Co-administration of fenugreek seed powder (200 mg/kg) was associated with partial attenuation of these adverse effects in the Pb co-exposure groups (50, 100, 150 mg/kg). At the matched Pb dose (G6 vs. G3, 150 mg/kg), co-treatment reduced hepatic and renal functional injury and lowered blood Pb by ~21%, supporting a genuine, if modest, fenugreek-associated effect. The progressively greater apparent protection at lower Pb co-exposure should be interpreted as a property of the co-exposure series rather than as a demonstrated fenugreek dose-response, given the absence of matched Pb-only comparators at 50 and 100 mg/kg. Renal protection was inferred from serum urea and creatinine and from blood Pb rather than from renal histology. Within the constraints of a single Pb-only reference dose and mRNA-level molecular endpoints, these preclinical findings warrant further investigation of fenugreek as a candidate protective adjunct against environmental Pb exposure, contingent on protein-level validation, pharmacokinetic characterization, and bioactivity-guided fractionation to identify the active constituent(s).

## Figures and Tables

**Table 3:** Chromatographic and spectral data and identification of compounds in fenugreek crude seeds.

Peak	Rt (min)	λmax (nm)	[M − H]^−^	Fragment Signals (*m*/*z*)	Compound Identification
1	9.25	228, 264	827	665, 545, 383, 341, 281, 221, 179, 146, 129, 110	Tricaffeoyl-glucosyl-glucoside
2	13.10	232, 276	695	619, 407, 363, 309,180, 167,128	Tricaffeoyl-hydroxyferulic acid
3	17.69	248	888	863, 452, 431, 171, 137	Dihydrogallic acid derivative
4	19.24	228, 260	947	765, 483, 382, 266, 205, 167, 115	Disynapoyl-hydro feruloyl-feruloyl-hydrocaffeic acid
5	20.29	236, 278, 328	447	224, 152, 136, 108	Galloyl-coumaric acid pentoside
6	21.42	230, 296	499	377, 273, 163, 119	Caffeoyl-coumaroyl-quinic acid
7	23.30	240, 334, 346	801	671, 477, 399, 323, 261, 144, 119	Dicaffeoyl-protocatechuic acid diglucoside
8	26.48	220, 234, 316	837	647, 625, 587, 452, 395, 347, 317, 293, 165, 132, 128, 115	Unidentified
9	31.70	234, 272, 334	593	473, 383, 353	Apigenin 6,8-di C-hexoside (vicenin 2 isomer)
10	32.30	234, 334	771	593, 503, 473, 383, 353	Apigenin 6-C-glucosyl 8-C-(2″-O-dihydroferuloyl)-glucoside
11	33.32	232, 272, 334	593	473, 383, 353	Apigenin 6,8-di C-glucoside (vicenin 2)
12	34.54	232, 270, 336	749	593, 503, 473, 383, 353	Vicenin derivative
13	35.09	232, 270, 336	593	473, 383, 353	Apigenin 6,8-di C-hexoside (vicenin 2 isomer)
14	38.18	232, 270, 336	563	443, 383, 353	Apigenin 8-C-xyloside-6-C-glucoside (vicenin 3)
15	39.59	234, 270, 348	895	563, 447, 357, 327, 284	Luteolin 7-O-[6″-dihydrogalloyl]-glucosyl-8-C-pentosyl-(1→6)-glucoside
16	40.41	270, 346	895	563, 447, 357, 339, 327, 285	Luteolin 7-O-[6″-dihydrogalloyl]-glucosyl-8-C-pentosyl-(1→2)-glucoside
17	41.57	230, 270, 336	563	443, 383, 353	Apigenin 6-C-xyloside-8-C-glucoside (vicenin 1)
18	44.28	232, 268, 336	863	563, 443, 323, 311	Apigenin 7-O-(2″-dihydrogalloyl)-rhamonsyl-6-C-(2‴-pentosy)-glucoside
19	46.13	232, 272, 338	577	503, 473, 383, 353	Apigenin 8-C-rhamnoside-6-C-glucoside
20	47.95	234, 270, 336	863	563, 443, 323, 311, 283	Apigenin 7-O-(2″-dihydrogalloyl)-rhamonsyl-6-C-(2‴-pentosy)-glucoside
21	49.68	232, 270, 336	725	533, 443, 413, 383, 353	Apigenin 6-C-pentosyl 8-C-(2″-O-hydroxyferuloyl)-pentoside
22	50.70	232, 272, 340	759	593, 473, 383, 353	Apigenin 6-C-glucosyl 8-C-(6″-O-methoxygalloyl)-glucoside
23	51.75	234, 270, 336	863	563, 431, 323, 283	Apigenin and 7-O-(6″-dihydrogalloyl)-rhamonsyl-6-C-(2‴-pentosy)-glucoside
24	53.3	232, 316	877	563, 473, 447, 327, 285	Kaempferol 7-O-(6″-galloyl)-glucosyl 6-C-(2‴pentosyl)-rhamnoside
25	55.52	232, 270, 346	877	533, 447, 357, 339, 305, 285	Luteolin 7-O-(2″-galloyl)-glucosyl 6-C-(2‴pentosyl)-rhamnoside
26	58.06	232, 270, 338	893	577, 473, 383, 353	Apigenin 7-O-(6″-dihydrogalloyl)-glucosyl-8-C-rhamnosyl-6-C-glucoside
27	60.09	230, 270, 338	893	577, 473, 383, 353	Apigenin 7-O-(2″-dihydrogalloyl)-glucosyl-8-C-rhamnosyl-6-C-glucoside
28	65.00	232, 270, 344	925	605, 563, 443, 383, 353	Luteolin 7-O-(6″-quinoyl)-rhamnosyl-6-C-pentosyl-8-C,O-(6‴acetyl)-glucoside
29	65.80	232, 270, 344	547	487, 457, 383, 353, 283	Luteolin 8-C-(2″-malonyl)-glucoside
30	68.86	270, 344	935	651, 547, 461, 327, 285	Luteolin 7-O-(2″dihydrogalloyl)-pentosyl-4′-O-(2‴,6‴-malonyl-pentosyl)-rhamnoside
31	77.12	232, 270, 316	593	447, 429, 309, 285	Kaempferol 7-O-rhamnosyl-(1→2)-glucoside
32	93.23	232, 270, 318	1133	1063, 917, 577, 164, 293	Kaempferol 7-O-(2‴,6‴,2″-malonyl)-rhamonsyl-diglucosyl-3-O-(6″″′rhamnosyl)-rhamnoside

**Table 4:** The quantitative chemical composition of the fenugreek crude seeds (the results are given in %).

Peak Number	Quantitative Chemical Composition (%)	Peak Number	Quantitative Chemical Composition (%)	Peak Number	Quantitative Chemical Composition (%)
1	0.18	12	0.68	23	0.27
2	0.19	13	5.67	24	0.82
3	3.14	14	14.46	25	0.09
4	0.47	15	3.78	26	0.04
5	0.44	16	15.90	27	0.39
6	0.24	17	8.80	28	0.43
7	0.36	18	6.56	29	0.42
8	0.73	19	1.16	30	0.61
9	0.29	20	12.1	31	8.71
10	0.81	21	0.32	32	2.50
11	9.66	22	0.32		

**Table 5:** Biweekly assessment of body weight (g) for the entire cohort of animals (n = 60).

Week	Mean	SD
W0	26.12	0.39
W2	27.1	0.72
W4	28.19	1.17
W6	29.21	1.64
W8	30.27	2.11

**Table 6:** Descriptive statistics of body weight (g) and daily food (g/mouse/day) and water intake (mL/mouse/day) across groups (n = 10 per group).

Groups	Body Weight		Food Intake (g/Mouse/Day)		Water Intake (mL/Mouse/Day)	
	Mean	SD	Mean	SD	Mean	SD
G1	32.38	0.52	5.059	0.039	12.02	0.395
G2	32.68	0.41	5.191	0.034	13.57	0.313
G3	26.65	0.37	4.548	0.036	7.858	0.291
G4	30.88	0.34	4.930	0.042	10.89	0.369
G5	30.07	0.33	4.821	0.035	9.938	0.337
G6	28.94	0.26	4.731	0.029	9.21	0.313

**Table 7:** Lead concentrations in blood (µg/dL).

Sample-ID	G1	G2	G3	G4	G5	G6
1	0.19	0.21	10.7	6.8	7.5	8.6
2	0.18	0.2	10.61	6.7	7.7	8.8
3	0.17	0.21	10.8	6.6	7.6	8.9
4	0.21	0.19	10.68	6.5	7.5	8.5
5	0.2	0.18	10.74	6.7	7.8	8.4
6	0.21	0.17	10.59	6.8	7.5	8.4
7	0.19	0.21	10.79	6.9	7.9	8.9
8	0.18	0.2	10.72	6.5	7.5	8.1
9	0.19	0.21	10.66	5.4	7.5	8.2
10	0.21	0.2	10.9	6.5	7.6	8.3
Mean	0.192	0.197	10.718	6.511	7.608	8.501

## Data Availability

All data generated or analyzed during this study are included in this published article. Additional datasets supporting the findings of this study are available from the corresponding authors upon reasonable request.

## Supplementary Materials

The following supporting information can be downloaded at: https://www.mdpi.com/article/10.3390/cimb48070650/s1.

## Abbreviations

The following abbreviations are used in this manuscript:

Pb	Lead
qRT-PCR	Quantitative Real-Time Polymerase Chain Reaction
ALT	Alanine Aminotransferase
AST	Aspartate Aminotransferase
ALP	Alkaline Phosphatase
Nrf2	Nuclear Factor Erythroid 2–Related Factor 2
HO-1	Heme Oxygenase-1
TNF-α	Tumor Necrosis Factor Alpha
IL-6	Interleukin 6
SCD-1	Stearoyl-CoA Desaturase-1
SOD-3	Superoxide Dismutase 3
BLLs	Blood Lead Levels
NIH	National Institutes of Health
PBS	Phosphate Buffered Saline
AAS	Atomic Absorption Spectrophotometry
H&E	Hematoxylin and Eosin
ELISA	Enzyme-Linked Immunosorbent Assay
