# Supplementary material for: Fenugreek Seed Powder Attenuates Lead-Induced Hepatic Injury and Renal Dysfunction in Male Mice Co-Exposed to Escalating Lead Doses
Source: Curr Issues Mol Biol. 2026 Jun 24;48(7):650. doi: 10.3390/cimb48070650 (PMC13406765; doi:10.3390/cimb48070650)
Supplement: Supplementary file 1 [file cimb-48-00650-s001.zip › cimb-4361510-supplementary.pdf]

Supplementary Table S1. Focused statistical analysis of all endpoints

Test selection was data-driven: Shapiro–Wilk (per group) for normality and Levene/Brown–Forsythe for variance homogeneity. One-way ANOVA (with Dunnett vs control and Šídák for co-exposure-vs-Pb contrasts) was used when assumptions held; Welch's ANOVA with Games–Howell when variances were heterogeneous; Kruskal–Wallis with Dunn's (Holm) when data were non-normal. Only the pre-specified, aim-relevant comparisons are shown (group/control). Cells give the significance symbol and adjusted p-value. n = 10 per group (body weight and all biochemical/molecular endpoints); n = 8 weekly cage-mean measurements per group for food and water intake. ns, not significant.

Endpoint	Omnibus test (post-hoc)	Statistic, p	Effect	G3/G1	G2/G1	G4/G3	G5/G3	G6/G3	G4/G1	G5/G1	G6/G1
**Body weight (W8)**	One-way ANOVA (Dunnett/Šídák)	F=352.9, p<0.0001	η^2=0.97	**** p<0.0001	ns p=0.2813	**** p<0.0001	**** p<0.0001	**** p<0.0001	**** p<0.0001	**** p<0.0001	**** p<0.0001
**Food intake**	One-way ANOVA (Dunnett/Šídák)	F=328.6, p<0.0001	η^2=0.98	**** p<0.0001	**** p<0.0001	**** p<0.0001	**** p<0.0001	**** p<0.0001	**** p<0.0001	**** p<0.0001	**** p<0.0001
**Water intake**	One-way ANOVA (Dunnett/Šídák)	F=291.4, p<0.0001	η^2=0.97	**** p<0.0001	**** p<0.0001	**** p<0.0001	**** p<0.0001	**** p<0.0001	**** p<0.0001	**** p<0.0001	**** p<0.0001
**ALT**	Kruskal–Wallis (Dunn, Holm)	H=54.2, p<0.0001	ε^2=0.91	**** p<0.0001	ns p=0.6726	** p=0.0025	ns p=0.0578	ns p=0.6482	ns p=0.1983	* p=0.0172	**** p<0.0001
**AST**	Kruskal–Wallis (Dunn, Holm)	H=54.2, p<0.0001	ε^2=0.91	**** p<0.0001	ns p=0.7392	** p=0.0011	ns p=0.1088	ns p=0.3702	ns p=0.1107	** p=0.0015	**** p<0.0001
**ALP**	Kruskal–Wallis (Dunn, Holm)	H=55.1, p<0.0001	ε^2=0.93	**** p<0.0001	ns p>0.999	** p=0.0014	ns p=0.0643	ns p=0.6609	ns p=0.1643	** p=0.0068	**** p<0.0001
**Urea**	Kruskal–Wallis (Dunn, Holm)	H=38.4, p<0.0001	ε^2=0.62	**** p<0.0001	ns p>0.999	ns p=0.052	ns p=0.5112	ns p>0.999	ns p=0.1517	** p=0.0065	*** p=0.0002
**Creatinine**	Kruskal–Wallis (Dunn, Holm)	H=55.3, p<0.0001	ε^2=0.93	**** p<0.0001	ns p=0.7978	** p=0.0012	ns p=0.0586	ns p=0.5811	ns p=0.2186	* p=0.0103	**** p<0.0001
**Blood lead**	Kruskal–Wallis (Dunn, Holm)	H=56.0, p<0.0001	ε^2=0.94	**** p<0.0001	ns p=0.7877	*** p=0.0007	* p=0.0413	ns p=0.3995	ns p=0.1187	** p=0.0042	**** p<0.0001
**Nrf2**	Welch's ANOVA (Games–Howell)	F=77.0, p<0.0001	η^2=0.89	**** p<0.0001	* p=0.0147	**** p<0.0001	**** p<0.0001	*** p=0.0008	** p=0.0093	**** p<0.0001	**** p<0.0001
**SOD-3**	Kruskal–Wallis (Dunn, Holm)	H=40.8, p<0.0001	ε^2=0.66	** p=0.0063	ns p=0.2564	**** p<0.0001	ns p=0.0696	ns p=0.2564	ns p=0.2423	ns p=0.4269	ns p=0.2847
**SCD-1**	Kruskal–Wallis (Dunn, Holm)	H=43.6, p<0.0001	ε^2=0.71	**** p<0.0001	ns p=0.1766	ns p=0.1766	**** p<0.0001	* p=0.0281	*** p=0.0002	ns p=0.1766	** p=0.0068
**HO-1**	Kruskal–Wallis (Dunn, Holm)	H=48.7, p<0.0001	ε^2=0.81	**** p<0.0001	ns p=0.5471	*** p=0.0004	ns p=0.0701	ns p=0.2956	ns p=0.5471	ns p=0.0637	** p=0.0078
**TNF-α**	Kruskal–Wallis (Dunn, Holm)	H=54.6, p<0.0001	ε^2=0.92	**** p<0.0001	ns p=0.5555	*** p=0.0008	ns p=0.1907	ns p=0.1907	ns p=0.2071	** p=0.0019	** p=0.0024
**IL-6**	Welch's ANOVA (Games–Howell)	F=250.8, p<0.0001	η^2=0.91	**** p<0.0001	** p=0.0012	**** p<0.0001	**** p<0.0001	*** p=0.0002	**** p<0.0001	**** p<0.0001	**** p<0.0001

Significance: *p<0.05, **p<0.01, ***p<0.001, ****p<0.0001. Values computed in Python (SciPy 1.17); methods are equivalent to the corresponding GraphPad Prism procedures and may differ from a Prism run only in the last reported decimal.
